# Location-Dependent Differences in Cardiac and Skeletal Muscle Dysfunction Associated With Truncating Titin (*ttn.2*) Variants

**DOI:** 10.1161/CIRCRESAHA.124.325999

**Published:** 2026-01-07

**Authors:** Celine F. Santiago, Inken G. Huttner, Ailbhe K. O'Brien, Melissa A.B. Amerudin, Pauline M. Bennett, Jasmina Cvetkovska, Renee Chand, Mark Holt, Gunjan Trivedi, Louis W. Wang, Xiaoping Yang, Kelly A. Smith, Mathias Gautel, Diane Fatkin, Yaniv Hinits

**Affiliations:** 1 Molecular Cardiology and Biophysics Division, Victor Chang Cardiac Research Institute, Darlinghurst, New South Wales, Australia (C.F.S., I.G.H., J.C., R.C., G.T., L.W.W., D.F.).; 2 School of Clinical Medicine, Faculty of Medicine and Health, UNSW Sydney, Kensington, New South Wales, Australia (C.F.S., I.G.H., L.W.W., D.F.).; 3 Randall Centre for Cell and Molecular Biophysics, and British Heart Foundation Centre of Research Excellence, King’s College London, United Kingdom (A.K.O, M.A.B.A., M.H., M.G., Y.H.).; 4 Randall Centre for Cell and Molecular Biophysics, King’s College London, United Kingdom (P.M.B.).; 5 CEMS Proteomics Facility, King’s College London, United Kingdom (X.Y.).; 6 Department of Anatomy & Physiology, School of Biomedical Sciences, University of Melbourne, Victoria, Australia (K.A.S.).; 7 Cardiology Department, St. Vincent’s Hospital, Darlinghurst, New South Wales, Australia (D.F.).

**Keywords:** cardiomyopathies, connectin (titin), muscular diseases, sarcomeres, zebrafish

## Abstract

**BACKGROUND:**

Truncating variants in the *TTN* gene (*TTN*tv), encoding the giant sarcomeric protein titin, cause a range of human cardiac and skeletal muscle disorders of varying penetrance and severity. The effects of variant location on clinical manifestations are incompletely understood.

**METHODS:**

We generated 6 zebrafish lines carrying truncating *ttn.2* variants in the Z-disk, I-band, A-band, and M-band titin regions. Expression of titin transcripts and protein levels was evaluated using quantitative polymerase chain reaction and proteomics. Phenotype analysis was performed during embryonic development and in adult hearts.

**RESULTS:**

Homozygous embryos from all lines except the C-terminal line, e232, showed a significant reduction of Z-disk and I-band *ttn.2* transcripts, but A-band and M-band transcript levels were reduced only in lines with truncations distal to the *cronos* promoter. These homozygous embryos uniformly died by 7 to 10 days postfertilization with marked impairment of cardiac morphology and function. Skeletal muscle motility and sarcomere organization were more disrupted in mutants with truncations distal to the *cronos* promoter compared with those proximal. In contrast, homozygous e232 embryos, which lacked only the titin kinase and M-band regions, had relatively preserved cardiac function with incorporation of truncated Ttn.2/Cronos protein and normal sarcomere assembly, but selective degradation of fast skeletal muscle sarcomeres. All heterozygous embryos were phenotypically indistinguishable from wild type. High-frequency echocardiography in adult heterozygous fish showed reduced ventricular contraction under resting conditions in A-band mutants. Heterozygous Z-disk and I-band mutants had no significant baseline impairment but were unable to augment ventricular contraction in response to acute adrenaline exposure, indicating a lack of cardiac reserve.

**CONCLUSIONS:**

Our data suggest that cardiac and skeletal muscle dysfunction associated with truncating *ttn.2* variants is influenced by age, variant location, and the amount of functional titin protein. The distinctive phenotype associated with distal C-terminal truncations may reflect different requirements for C-terminal titin for maintenance of fast, slow, and cardiac muscle sarcomeres.

**Novelty and Significance**

**What Is Known?**

- *TTN*tv are the most common genetic factor in dilated cardiomyopathy.
- Most reported dilated cardiomyopathy-associated *TTN*tv occur in the titin A-band.
- Childhood-onset skeletal myopathy is typically associated with compound heterozygosity or homozygosity for *TTN*tv in various domains.

**What New Information Does This Article Contribute?**

- *TTN*tv in constitutively expressed exons result in overt or latent cardiac dysfunction, except for variants in the M-band.
- The propensity for skeletal muscle dysfunction varies with *TTN*tv location, proximal or distal to the *cronos* promoter.
- *TTN*tv lacking the titin kinase and M-band regions show selective degradation of fast skeletal muscle fibers and appear to act by a distinct mechanism.

In patients with dilated cardiomyopathy, determining the clinical significance of non-A-band *TTN*tv is challenging. Further, why some *TTN*tv show a predilection for cardiac or skeletal muscle dysfunction is unclear. Using a series of zebrafish models, we showed distinctive differences in the effects of *TTN*tv according to age, variant location, and levels of functional titin protein. Overall, our data support haploinsufficiency as a disease mechanism. However, the distinctive phenotype associated with M-band *TTN*tv is most readily explained by dominant negative effects of persistent truncated protein and highlights unique developmental and functional properties of this region. Our data will inform the interpretation of clinical genetic testing.

**Meet the First Author, see p e000745**

Titin is a giant protein that spans half-sarcomeres from the Z-disk to the M-band in the heart and skeletal muscle. It is a key determinant of passive muscle stiffness and active contraction in health and disease.^1^ In recent years, clinical interest in titin has escalated due to discoveries of genetic variants in patients with cardiac and skeletal myopathies. Truncating variants in the *TTN* (titin) gene (*TTN*tv) are now known to be the most common genetic risk factor for dilated cardiomyopathy (DCM).^2,3^ Although *TTN*tv-associated DCM is typically a dominant adult-onset condition, albeit with variable penetrance, individuals who have 2 abnormal *TTN* alleles leading to truncated proteins (compound heterozygotes or homozygotes) present with childhood-onset skeletal myopathy and cardiomyopathy if both variants are in cardiac-expressed exons.^4–8^ *TTN*tv also cause cardiac and skeletal myopathy in compound heterozygosity with destabilizing titin missense mutations.^5,7^ *TTN*tv have been reported in up to 3% of the general population^2^ and frequently arise as incidental findings in genetic testing for unrelated disorders.

Effects due to variant location have been proposed as a useful way to stratify *TTN*tv. Most DCM-associated *TTN*tv are situated in the titin A-band region, while skeletal muscle-associated variants are distributed across the gene.^2–4,9^ This simplistic view does not account for the presence of *TTN*tv in non-A-band locations in patients with DCM, nor A-band *TTN*tv in healthy subjects. Although most patients with A-band *TTN*tv have cardiac presentations, there are increasing reports of overt or subclinical skeletal muscle involvement.^10^ Further, children with congenital recessive *TTN*tv-associated skeletal myopathy may progressively develop DCM.^4,8^ There are numerous developmental and tissue-specific titin isoforms that arise due to complex alternate splicing. The extent to which any single exon is represented across the range of titin isoforms is indicated by the percent spliced-in (PSI) score. DCM-associated *TTN*tv are thought to be preferentially distributed in exons that have high PSI scores in the adult heart.^3^ Whether PSI scores are useful in the context of skeletal myopathy has yet to be clarified. Identifying the subset of *TTN*tv with the greatest risk of pathogenicity is, therefore, crucial for informed medical management and genetics counseling of patients and their families.

Zebrafish are a useful animal model to study *TTN*tv effects. There are 2 titin genes in zebrafish, *ttn.2* (*ttna*) and *ttn.1* (*ttnb*). During early development, *ttn.2* is expressed in both cardiac and skeletal muscle and is the primary titin gene in the heart.^11^ Both heart and skeletal muscle phenotypes have been reported in homozygous *ttn.2* mutants.^12^ We have previously reported adult-onset DCM in heterozygous *ttn.2* zebrafish generated to carry a human A-band *TTN*tv.^13^ *ttn.1* is mainly expressed in skeletal muscle, and *ttn.1* mutants have been reported to have predominant skeletal myopathy phenotypes.^14,15^ An alternative promoter in *ttn.2* encodes Cronos, a short titin isoform comprising A-band and M-band domains. First discovered in zebrafish, the Cronos isoform is conserved in mouse and human.^16,17^ Cronos transcripts are abundant in skeletal muscle, but low in developing hearts.

The aim of our study was to investigate the impact of variant location on cardiac and skeletal muscle structure and function in embryonic and adult zebrafish, using a series of mutant lines carrying *ttn.2* variants predicted to truncate Z-disk, I-band, A-band, and M-band regions.

## Methods

### Data Availability

Data are available from the corresponding authors on reasonable request. Expanded Methods and a Major Resources Table are provided in the Supplemental Material.

### Zebrafish Husbandry

Zebrafish (Danio rerio) were raised according to standard procedures.^18^ Experiments using zebrafish were performed in compliance with relevant laws and institutional guidelines in Australia and the United Kingdom.

### Generation of Zebrafish Lines

**Figure 1.**
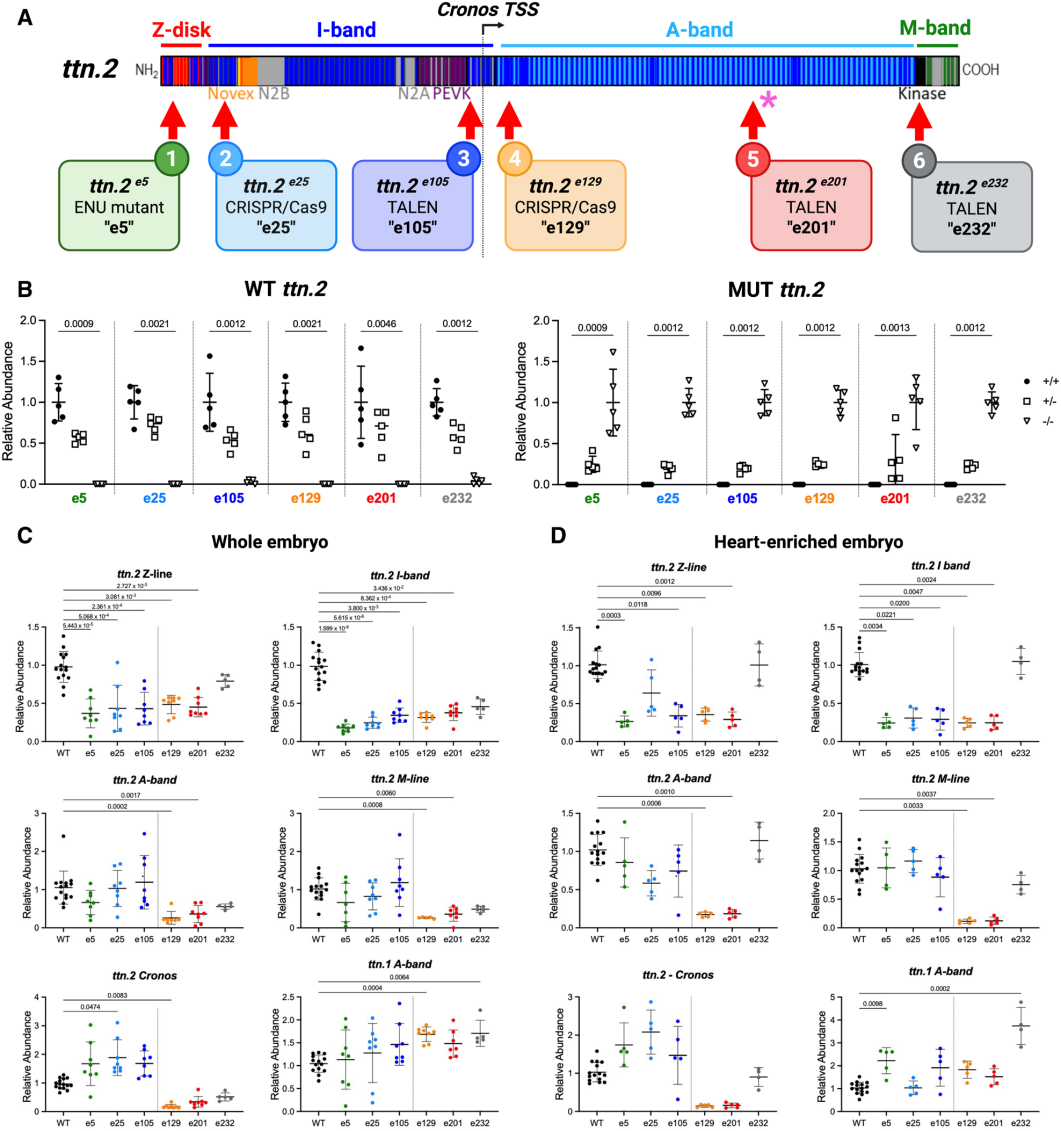
**Zebrafish *ttn.2* truncating variants. A**, Schematic of the Ttn.2 protein showing major domains and the *cronos* translation start site (TSS). Locations of *ttn.2* truncations (red arrows) and the *ttn*^*x071*^ mutant^14^ (asterisk) are indicated. **B**, Quantitative polymerase chain reaction (qPCR) analysis of homozygous (−/−), heterozygous (+/−), and wild type (WT; +/+) whole embryos using primers specific for WT (**left**) and mutant (**right**) alleles; n=5/genotype. **C**, qPCR analysis of different *ttn.2* and *ttn.1* regions in WT (n=15) and homozygous (n=8 for all lines except e232, where n=5) whole embryo. **D**, qPCR analysis of WT (n=15) and homozygous (n=5 for all lines except e232, where n=4) heart-enriched embryonic samples. qPCR data were normalized to house-keeping genes *tmem50a* and *ube2a* and expressed as transcript abundance relative to wild type levels. Data were analyzed using Scheirer-Ray-Hare analysis (**B**) or Kruskal-Wallis test (**C** and **D**) with post hoc Dunn multiple comparisons test (**B**: 18 comparisons; **C** and **D**: 6 comparisons). *P* values were adjusted for multiple comparisons testing using the Bonferroni correction. mean±SD.

Zebrafish lines carrying truncating mutations in 6 different locations in the *ttn.2* gene and 1 in *ttn.1* were generated (Figure 1A; Figures S1 and S2; Expanded Methods (Supplemental Material). The *ttn*^*xu071*^ line (kindly provided by Xiaolei Xu) has a double truncation (*ttn.2* A-band and *ttn.1* Z-disk).^14^ Genotyping was performed by polymerase chain reaction (PCR) amplification followed by Sanger sequencing or restriction enzyme digest, using primers and enzymes listed in Table S1.

### qRT-PCR

Total RNA from whole embryos (30 embryos/sample, n=5 samples, 3–5 dpf), heart-enriched (dissected) embryos (25 embryos/sample, n=5 samples, 3–5 dpf), or pooled wild type (WT) and heterozygous *ttn.2* adult hearts (2 hearts/sample, n=5 samples) was extracted using TRIzol (Sigma-Aldrich, St. Louis, MO) and the RNeasy Micro Kit (Qiagen, Hilden, Germany).^19^ Purified RNA was used to generate cDNA using the Superscript III First-Strand Synthesis System (Invitrogen, Burlingame, CA). Relative quantitative PCR (qPCR) was performed using a Light Cycler 480 thermal cycler (Roche, Basel, Switzerland) with primers listed in Table S2. Gene expression was normalized to the expression level of the housekeeping genes *tmem50a* and *ube2a* using ΔΔCt (cycle threshold) values and graphed relative to transcript expression in WT fish or as absolute expression.

### In Situ mRNA Hybridization and Immunofluorescence

In situ mRNA hybridization was performed as described.^20^ Probes used were kinase, N2A, and N2B for *ttn.2* and *ttn.1*, and *cronos*^16^ (primers listed in Table S3). For immunohistochemistry, primary antibodies were α-actinin (A7811; Sigma-Aldrich), actin (Actc1a, GeneTex, Irvine, CA), MyBP-C (myosin binding protein C),^21^ an antibody made against human cardiac C0-C1 that recognizes cardiac, slow (strongly) and fast (weakly) skeletal isoforms in zebrafish, Myom1 (Myomesin 1 B4^22^), Myom2 (Myomesin 2; M-protein, AA259),^23^ sarcomeric MyHC (myosin heavy chain; A4.1025),^24^ fast MyLC (myosin light chain; F310),^25^ titin (T12),^26^ titin (Z1Z2),^27^ Ttn.2 (Ttn.2 M6, see below), Ttn.1 (Ttn.1 M8-M9, see below). Secondary antibodies included Alexa dye-conjugated (Thermo Fisher Scientific), Goat anti IgA FITC (F9384, Sigma-Aldrich), and Cy3 AffiniPure Goat Anti Mouse IgG, Fcγ fragment specific (Jackson ImmunoResearch Laboratories). Samples were fixed and stained as described^28^ and imaged on a Zeiss LSM510.

### Antibody Production and Testing

To generate specific antibodies to the zebrafish Ttn.1 and Ttn.2 M-band, we identified unique regions from sequence alignments (Table S4). The M6 Ttn.2 antibody was designed for a region that shares high homology to human M6 but is missing from Ttn.1. The M8-M9 Ttn.1 antibody was designed for a region homologous to the human Mis6-M8-M9-Mis7 region in Ttn.1. Protein fragment preparation is described in the Extended Methods (Supplemental Material). Rabbits were immunized with protein fragments, and polyclonal sera were collected (Eurogentec).

### Titin Protein Gels and Western Blots

Titin isoforms in embryo tails (n=20 tails per biological replicate) were resolved in 1% SDS-SeaKem Gold agarose (Lonza) gels^29^ and visualized on a ChemiDoc MP system (Bio-Rad). The relative optical density of titin bands was calculated from n=3 gels per experiment. Western blotting was performed using anti-titin antibodies Z1Z2^8^ and Ttn.2 M6. Hearts (ventricle and atrium only) from adult male WT and heterozygous *ttn.2* zebrafish (≈14 months old, n=2 per sample).^30^ Following SDS- polyacrylamide gel electrophoresis, gels were either stained immediately using Coomassie blue to visualize total protein or transferred onto 0.2 µm PVDF membranes, which were then immunostained for titin using anti-*TTN* mouse monoclonal antibody (2F12, Abnova, No. H00007273-M07A) or anti-mouse HRP (Cytiva, No. NA9310).

### Proteomics Analysis

Whole zebrafish 3 dpf embryos lysates were lysed within 6M urea buffer containing 5.4% SDS, 4.45% β-mercaptoethanol, 2.3% NP-40, 136.4 mmol/L Tris (pH 6.8) with protease inhibitor and phosSTOP. Samples processing and mass spectrometry analysis are described in the Extended Methods (Supplemental Material). The mass spectrometry proteomics data have been deposited to the ProteomeXchange Consortium via the Proteomics Identification Database^31^ partner repository with the project accession number: PXD071430.

### Electron Microscopy

Embryos were fixed in a solution of 4% paraformaldehyde/2.5% glutaraldehyde in PBS for 1 hour on ice, postfixed in a 1% osmium tetroxide in PBS solution for 30 minutes on ice, followed by graded dehydration in ethanol on ice. Sections were stained with uranyl acetate or UranyLess (Electron Microscopy Services, Hatfield, PA). Electron microscopy was carried out using a JEOL JM1400 transmission electron microscope in the Center for Ultrastructural Imaging, King’s College London.

### Video-Microscopy

Phenotypic evaluation was performed in anesthetized zebrafish embryos at room temperature using video-microscopy as described.^13^ Heart rates and fractional area change of the ventricle and atrium were obtained from the video images.

### High Frequency Echocardiography

High-frequency echocardiography was performed in adult male zebrafish aged 9 to 15 months using the Vevo3100 Imaging Station (VisualSonics, Amsterdam, the Netherlands) as described.^13,32^ Image analysis was performed using the VevoLab analysis software package version 5.7.0 (VisualSonics)^13,32^ by a single operator who was blinded to genotype. Parameters of cardiac size and contractile function were assessed.

### Adrenaline Stress

Ten- to 15-month-old heterozygous e5 (e5+/−) and e105 (e105+/−) mutants and WT siblings were subjected to acute adrenaline stress by submersion in 500 µmol/L epinephrine hydrochloride (E4642, Sigma-Aldrich) for 2 hours.^33^ Systolic and diastolic function were measured at baseline and at the conclusion of adrenaline exposure using high-frequency echocardiography.

### Statistical Analysis

Statistical analyses were performed with GraphPad Prism (GraphPad Software Inc, CA) unless otherwise specified. Normality testing was performed using the Shapiro-Wilk method. For data that were not normally distributed or for sample sizes that were small (<10/group), comparisons between groups were made using Kruskal-Wallis or Scheirer-Ray-Hare tests with adjustment for interactions and multiple comparisons. Data points in plots represent biological replicates, with bars representing mean±SD unless otherwise specified. Significance level (α) was *P*≤0.05 for all studies.

## Results

### *ttn.2* Zebrafish Models

We generated a series of 6 zebrafish *ttn.2* lines carrying variants predicted to result in a truncated titin protein (Figure 1A; Figure S1), with our nomenclature referring to the exon number of the truncation (with e1 being exon 1, etc). The e5 line has a nonsense mutation that introduces a premature stop codon in exon 5 before the Z-repeats in the proximal Z-disk region. The e25 line has a 2 bp insertion and a premature stop codon in exon 25 in the proximal I-band. In the e105 line, a 7 bp deletion in exon 105 is predicted to shift the reading frame with 12 residues of neo-sequence followed by a premature stop codon at the amino acid corresponding to human *TTNtv*, p.Y12304*. Exon 105 is in the distal I-band proximal to the *cronos* promoter. The e129 line has a 5 bp deletion and stop codon in exon 129 distal to the *cronos* promoter near the I-band/A-band junction. The e201 line, modeling the human *TTN*tv p.R26331*, has previously been described^13^ and carries an 8 bp deletion and stop codon in exon 201 in the mid A-band. The e232 line has a 1 bp deletion and an 8 bp insertion in exon 232 in the distal A-band, upstream of the titin kinase domain and M-band.^34^ Zebrafish exons targeted in these mutants have high sequence homology to the corresponding human *TTN* exons, all of which have high PSI scores in the adult human heart (Table S5).

WT and mutant *ttn.2* alleles were quantified by qPCR analysis of pooled whole embryos (3–5 dpf). Using WT allele-specific primers, levels of WT *ttn.2* transcript were reduced by ≈50% in heterozygous (+/-) mutants and absent in homozygous (−/−) mutants (Figure 1B, left panel). Reciprocal patterns were seen using mutant allele-specific primers (Figure 1B, right panel).

Abundance of titin transcripts was evaluated using location-specific primers in whole embryo (Figure 1C) and in heart-enriched embryonic tissue samples (Figure 1D). In both types of embryonic tissue preparation, e5−/−, e25−/−, and e105−/− embryos showed reduction of Z- and I-band *ttn.2* transcripts while A- and M-band transcripts were detected at normal levels. In contrast, e129−/− and e201−/− embryos had a reduction of detectable *ttn.2* transcripts across all domains. We hypothesized that the differences might be explained by differential upregulation of the *ttn.2* *cronos* isoform.^16^ Indeed, there was a trend towards increased expression of *cronos* transcripts in e5−/−, e25−/−, and e105−/− embryos (proximal to the *cronos* promoter) but not in e129−/− and e201−/− (distal to the *cronos* promoter) in both whole embryo and heart-enriched samples. This may reflect upregulation of *cronos* in response to mechanisms triggered by degraded mutant mRNA.^35^ The transcript profiles in e232−/− embryos, including *cronos*, were not significantly different from WT. *ttn.1* levels were modestly increased in most lines but showed marked upregulation in the heart-enriched e232−/− samples.

### Ttn protein Levels Are Downregulated in Mutants

**Figure 2.**
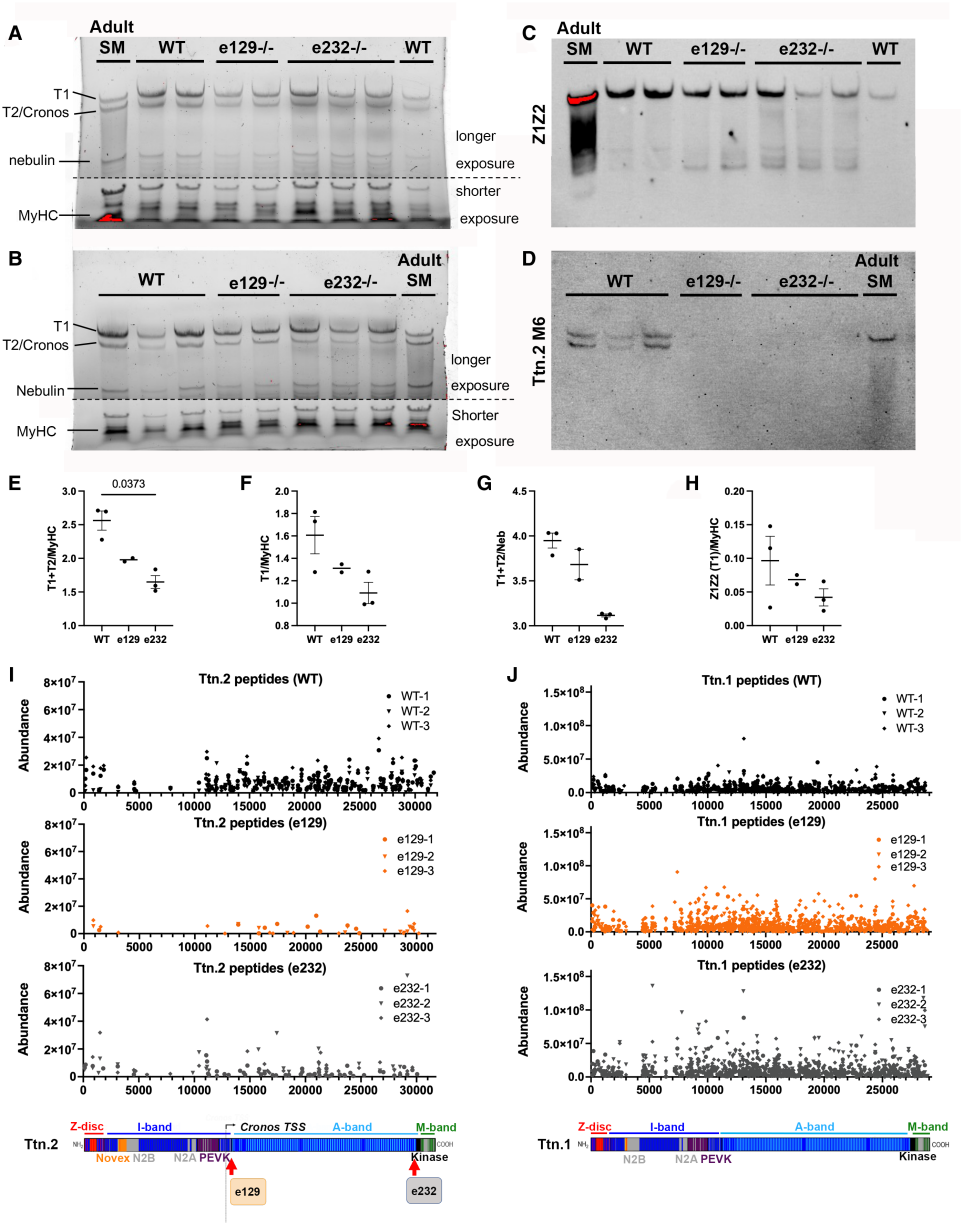
**Ttn (titin) protein levels in homozygous *ttn.2* mutant embryos. A** and **B**, Images of in-gel stained 1% agarose gels of 5 dpf wild-type (n=3), e129−/− (n=2) and e232−/− (n=3) embryo tail samples and an adult skeletal muscle (SM) sample for comparison. Major proteins are indicated. The lower part of the gels is shown at a shorter exposure. **C** and **D**, Gels from **A** and **B** immunoblotted with titin Z1Z2 antibody (**C**) or with Ttn.2 M6 antibody (**D**). **E** through **G**, Ttn relative abundance is expressed as normalized Ttn total protein (T1+T2) band intensity divided by MyHC (myosin heavy chain) intensity (**E**), T1 band intensity divided by MyHC (**F**; T1+T2) band intensity divided by Nebulin band intensity (**G**), or Z1Z2 band intensity divided by MyHC intensity (**H**). **I** and **J**, Proteomics analysis showing Ttn.2 (**I**) and Ttn.1 (**J**) peptide abundance in 3 to 5 dpf wild type, e129−/− and e232−/− embryos plotted against the amino acid start position of detected peptides in Ttn.2 (Uniprot accession A0AB32TUY4) and Ttn.1 (Uniprot accession A0AB32TZZ1) shown above schematic of Ttn.2 and Ttn.1 proteins, respectively. Statistical comparisons were performed using the Kruskal-Wallis test with Dunn multiple comparison test (3 comparisons), *P* values adjusted for multiple comparisons using Bonferroni correction, mean±SEM.

Assessment of titin protein was undertaken in 2 mutants, e129−/− and e232−/−, that have distinct mRNA profiles and phenotypes (see below). In-gel-stained 1% agarose gels were used to separate the titin species (Figure 2A and 2B). Gels were then immunoblotted with antibodies upstream (Z1Z2) and downstream (Ttn.2 M6; Figure S2) from the truncation position (Figure 2C and 2D). Two major bands were detected in all samples: the T1 full-length Ttn.1 and Ttn.2 proteins, and the smaller T2, assumed to reflect a titin proteolytic degradation product and possibly the Cronos isoform (Figure 2A and 2B). Densitometry quantification of the total titin (T1+T2) or T1 alone to other bands on the same gel, such as MyHC or nebulin (Figure 2E through 2G) showed a significant reduction in titin levels in both e129−/− and e232−/− embryos compared with WT embryos. Z1Z2 antibody, which can react with both Ttn.1 and Ttn.2, detects only the full-length T1 titin, and when quantified, shows a similar trend (Figure 2C and 2H). Ttn.2 M6 is specific for Ttn.2 and binds to both T1 and T2 species in WT samples; no Ttn.2 was detected downstream of the respective truncations. No smaller-sized titin could be detected. This could be either because the truncated Ttn.2 protein is largely degraded, as the proteomic quantification below may suggest, or because resolution of our titin gels, especially in e232−/− samples (expected to be ≈95% of full size), is not sufficient to distinguish from full-size Ttn.1.

Since quantification of titin by comparison to other muscle proteins could be affected by concerted downregulation, proteomics analyses were conducted in 5 dpf embryos of the same groups, that is, WT, e129−/−, and e232−/−. A total of 2415 proteins was identified across all 3 experimental groups (full data in Proteomics Supplemental Files 2 and 3, and EMBL-EBI PRIDE [Proteomics IDEntifications Database] project PXD071430). Data for Ttn.2 and Ttn.1 are shown here (Figure 2I and 2J). Compared with WT, e129−/− Ttn.2 levels were reduced 3.1-fold and showed only a few sporadic Ttn.2 peptides in low abundance (over 10-fold decrease) clustering mainly in A-band titin (12.6±1.2 Ttn.2 peptides in average compared with 145±5 peptides in WT), whereas peptides of Ttn.1 were upregulated throughout (2.55-fold upregulated and 295.6±37 Ttn.1 peptides in average compared with 287.8±16.8 peptides in WT; Figure 2I and 2J and Proteomics Supplemental Files 2 and 3), mirroring our qPCR findings (Figure 1C and 1D). Ttn.2 levels in e232−/− samples were reduced overall to about 85% of WT protein, and 38±14 Ttn.2 peptides on average, compared with 145±5 peptides in WT, but did include some peptides distributed throughout the protein up to the truncation site just before the kinase. Ttn.1 peptides were also upregulated in e232−/− (3.1-fold upregulated and 286.3±77.7 Ttn.1 peptides on average compared with 287.8±16.8 peptides in WT; Figure 2I and 2J; Proteomics Supplemental Files 2 and 3). Taken together, the results indicate that there is no significant Ttn.2 protein in e129−/−, whereas truncated Ttn.2 is detectable in e232−/− but at much lower levels. In both mutants, Ttn.1 levels were upregulated, presumably as a compensatory response.

### Embryonic Cardiac Phenotypes

**Figure 3.**
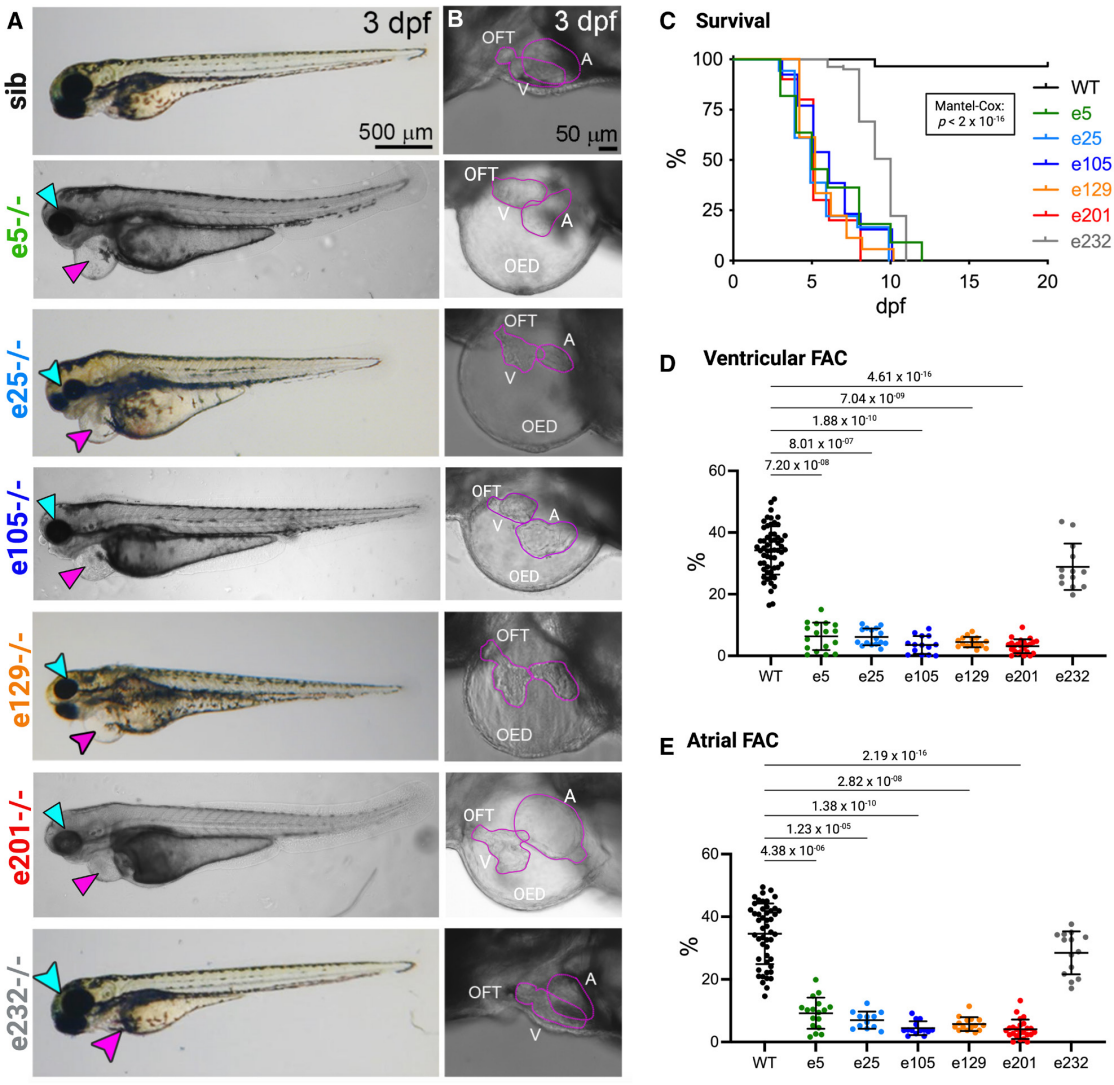
**Cardiac morphology and function in homozygous *ttn.2* embryos. A**, Brightfield images of 3 dpf homozygous and sibling control embryos shown in lateral view, anterior to left, with (**B**) higher magnification of the heart. With the exception of e232−/−, mutant embryos show pericardial edema (magenta arrowhead), as well as small eyes and head (cyan arrowhead). **C**, Survival in homozygous (e5, n=11; e25, n=18; e105, n=13; e129, n=18; e201, n=10; e232 n=81) and wild type (WT, n=29) embryos between 0 and 20 dpf; Mantel-Cox test. Ventricular (**D**) and atrial (**E**) contractile function in WT (n=57) and homozygous (e5, n=17; e25, n=16; e105, n=14; e129, n=15; e201, n=24; e232, n=13) embryos were assessed using video-microscopy. Data are expressed as fractional area change (FAC; %). Kruskal-Wallis test with Dunn multiple comparisons (6 comparisons), *P* values adjusted for multiple comparisons with Bonferroni correction, mean±SD. A indicates atrium; OED, edema; OFT, outflow tract; and V, ventricle.

All in-crosses of heterozygous alleles gave rise to Mendelian-ratio progenies. Homozygous embryos from e5, e25, e105, e129, and e201 lines were visually identified from 24 hpf by a dent in the yolk caused by pericardial effusion shortly after the heart tube begins to contract at 23 hpf.^36^ By 3 dpf, there was severe pericardial edema, an indicator of impaired ventricular contraction, as well as small eyes and craniofacial dysplasia (Figure 3A and 3B). The heart chambers were small and linearly arranged, indicating poor development and impaired looping (Figure 3B). e232−/− embryos were a notable exception, with overall WT appearance and cardiac morphology. Homozygous e5, e25, e105, e129, and e201 embryos died between 4 and 10 dpf (Figure 3C); e232−/− mutants died as larvae but survived a few days longer than other lines. There were no differences in survival between heterozygotes and WT siblings.

Cardiac function was evaluated in embryonic fish using video-microscopy as described in the Methods section. For all lines except e232, homozygous mutants showed a marked reduction in ventricular and atrial contractile function (assessed by fractional area change) from 3 dpf onwards (Figure 3D and 3E). Cardiac function in heterozygous *ttn.2* embryos was indistinguishable from WT siblings (Figure S3). Heart rates were similar in WT and mutant embryos.

### Myocardial Architecture in Embryonic Fish

**Figure 4.**
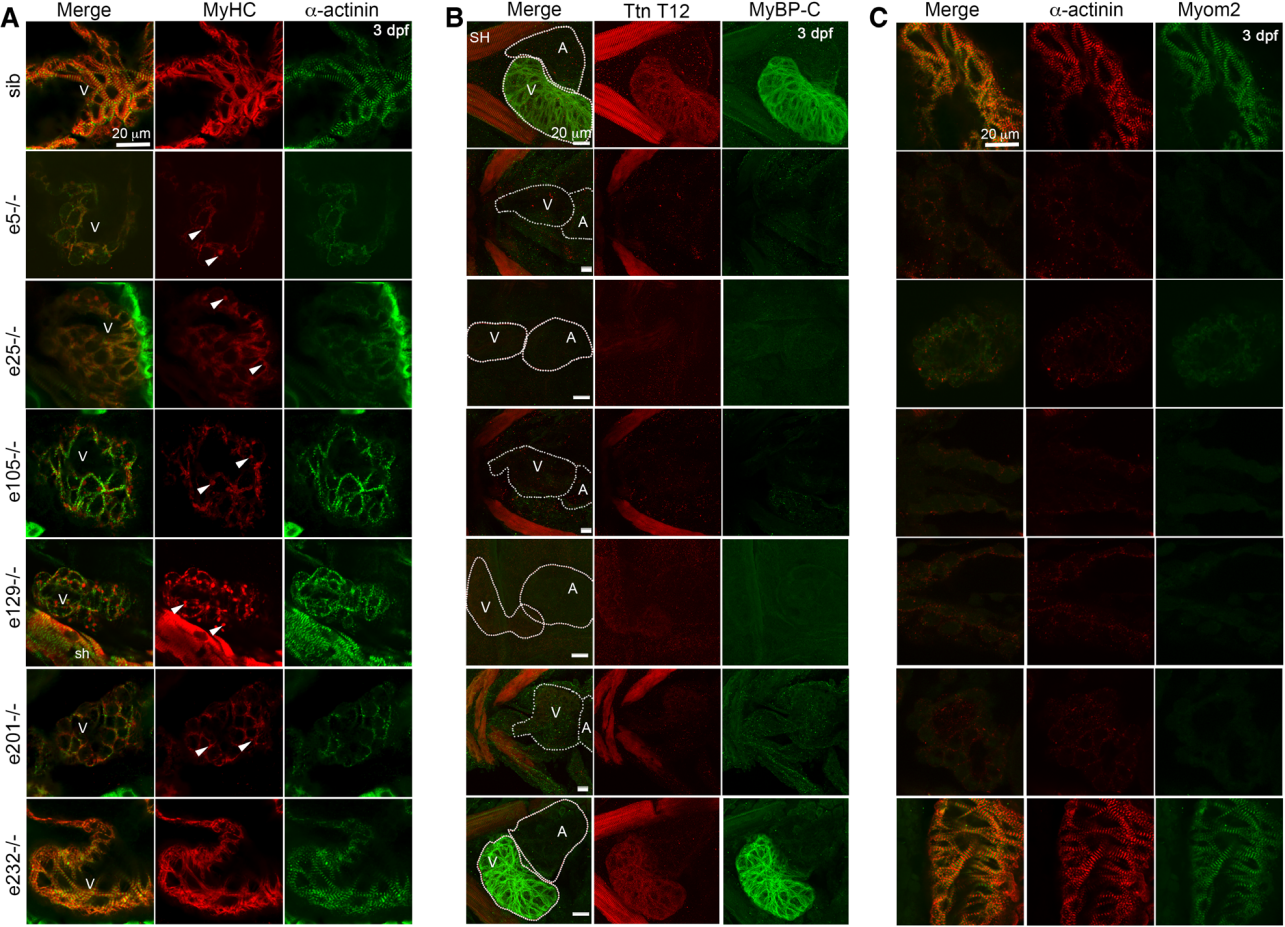
**Cardiac sarcomere assembly in *ttn.2* embryos. A**, Single confocal scans through ventricular sarcomeres of 3 dpf homozygous and sibling (sib) control embryos, immunostained for MyHC (myosin heavy chain) and α-actinin. Mutants show punctate aggregates (arrowheads) except e232−/− embryos in which sarcomeres appear normal. **B,** Confocal stacks of 3 dpf hearts of homozygous *ttn.2* and sibling control embryos, immunostained for Ttn (titin) T12 and MyBP-C (myosin binding protein-C), shown in ventral view, anterior to left. Heart chambers show very low levels of Ttn T12, and MyBP-C. **C**, Single confocal scans through ventricular sarcomeres of homozygous mutants and sibling control embryos, immunostained for α-actinin and Myom2 (myomesin-2). Mutant embryos show punctate aggregates of α-actinin and only low levels of Myom2. In **B** and **C**, cardiac morphology and sarcomeres in e232−/− embryos are similar to control. A indicates atrium; SH, sternohyoideus muscle; and V, ventricle.

Embryonic hearts were evaluated using immunofluorescence staining. At 3 dpf, the intensity of MyHC staining in e5−/−, e25−/−, e105−/−, e129−/−, and e201−/− embryonic hearts was reduced, with numerous aggregates and few very disorganized fibrils (Figure 4A; Figure S4A and S4B). In WT embryos, there was strong staining using the T12 antibody recognizing an epitope in I2-I3 at the start of the I-band in both Ttn.2 and Ttn.1^37^ (Figure 4B), as well as with Ttn.1 M8-M9 antibody that can detect both Ttn.1 and Ttn.2 (Figure S5; Figure S2A and S2C). T12 and Ttn.1 M8-M9 immunostaining in e5−/−, e25−/−, e105−/−, e129−/−, and e201−/− embryonic hearts show that titin is diminished (Figure 4B; Figure S4C). Z-disk titin interacts with the cytoskeletal protein, α-actinin.^38^ In e5−/−, e25−/−, e105−/−, e129−/−, and e201−/− embryos, α-actinin staining showed aggregates and poorly organized striations, suggesting that some Z-I brushes may have formed despite the depletion of titin (Figure 4A and 4C; Figure S4C). MyBP-C, which is in the C-zone of A-band titin,^34^ was absent from the cardiac sarcomeres of these mutants (Figure 4B). Despite the apparent absence of direct titin-MyBP-C contacts in the assembled (relaxed) thick filaments,^34^ these findings suggest that A-band titin is indispensable for the correct formation of the myosin-MyBP-C complex in vivo. Myom2 interacts with titin in the M-band in normal hearts (reviewed in Lange et al^39^). Myom2 was disorganized and present only at very low levels, suggestive of impaired M-band myosin-titin arrays (Figure 4C).

Cardiac expression of Ttn.2, Ttn.1, and Cronos were evaluated using in situ hybridization and immunostaining. Intriguingly, Ttn.1 M8-M9 showed normal cardiac staining but reduced M-band staining in somitic muscle sarcomeres of *ttn.1* e7−/− embryos. In WT embryos, *ttn.1* transcripts are expressed in the heart at 24 hpf (Figure S6B, S6D, and S6F and Seeley et al^11^), but expression becomes undetectable by 3 dpf (Figure S6H, S6J, and S6L). Skeletal muscle expresses both Ttn.1 and Ttn.2 at these time points (Figure S6M through S6X). Like previous studies,^14^ we found that embryonic heart development in *ttn.1* mutant lines is normal (Figure S5A through S5C′). These findings suggest Ttn.2 but not Ttn.1 is essential for normal sarcomere assembly and formation of a functional heart. Our qPCR data in whole embryos (Figure 1C) suggested that *cronos* upregulation might be a potential compensatory mechanism for titin deficiency. However, these changes appear to be driven primarily by *cronos* expression in skeletal muscle, since qPCR for enriched-heart samples shows no significant upregulation of *cronos* transcript (Figure 1D) and in situ hybridization failed to detect *cronos* in the heart (Figure S6Y and S6Z and Zou et al^16^).

In keeping with preserved *ttn.2* transcript levels and cardiac function, sarcomere organization was indistinguishable in e232−/− embryos compared with WT siblings (Figure 4A through 4C; Figure S4A through S4C). We speculate that e232 transcripts may be incompletely degraded and that the truncated e232 Ttn.2 protein (Figure 2) is incorporated into cardiac sarcomeres and able to facilitate near-normal organization of myosin and other sarcomeric proteins, including M-band proteins, such as Myom2.

### Skeletal Muscle Embryonic Phenotypes

**Figure 5.**
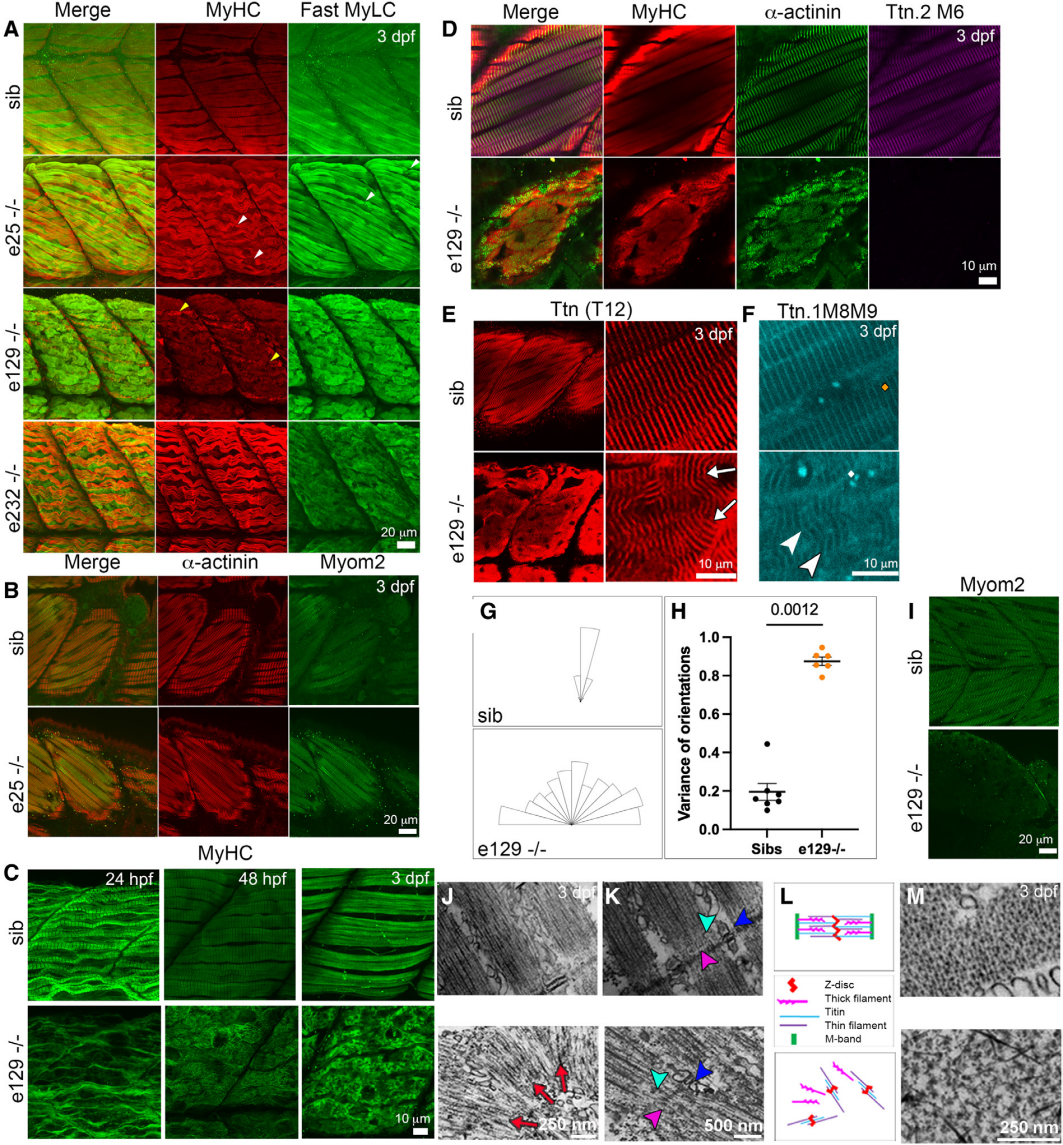
**Skeletal muscle phenotypes in *ttn.2* embryos. A** through **G** and **I**, Immunofluorescence staining of somitic muscle of 3 dpf homozygous and sibling (sib) control embryos for MyHC (myosin heavy chain), Fast MyLC (fast myosin light chain), α-actinin, Myom2 (myomesin-2), Ttn.2 M6, Ttn T12, and Ttn.1 M8-M9, shown in lateral view, anterior to left. **A**, MyHC is strongly expressed in superficial slow fibers, while MyLC labels fast fibers. e25−/− embryos show some good sarcomeric structure as well as regions of damaged sarcomeres in the middle of the fiber (white arrowheads). e129−/− embryos have disorganized sarcomeres in both slow (yellow arrowheads) and fast fibers, while e232−/− embryos have striated slow fibers but no organized thick filaments in fast fibers. **B**, In e25−/− embryos, α-actinin (slow and fast fibers) and Myom2 (fast only) seem striated. **C** through **I**, In e129−/− embryos, abnormal striation is detected with MyHC from 24 hpf to 3 dpf. MyHC and α-actinin show no normal striation but instead have radial organization around random points in the fiber, and no Ttn.2 M6 (**D**). This abnormal striation is also seen using Ttn T12 (**E**) and Ttn.1 M8-M9 (**F**), Myom2 is abolished in e129−/− embryos (**I**). **G** and **H**, Quantification of directionality of fibers. Rose diagram of representative somitic muscle (**G**) and graph comparing the variance of the orientations (**H**). The possible range is 0 to 1, where 0 is completely ordered and 1 is completely random. Unpaired Mann-Whitney *U* test, mean±SEM (sibs n=7, e129−/− n=6). **J** through **M**, Electron micrographs of longitudinal (**J** and **K**) or transverse (**M**) sections of 3 dpf e129−/− and sibling control embryos. Thin filaments in mutant embryos are arranged in I-Z-I brushes that are organized in radiating patterns (**J**, red arrows), highlighting structural features (**K**), including triads (blue arrowheads), Z-disks (pink arrowheads), and thin filaments (cyan arrowheads). Schematic illustrating the arrangement of thick and thin filaments (**L**). e129−/− embryos show a changed lattice pattern of thick and thin filaments with increased interfilament space compared with sibling controls (**M**).

Skeletal muscle motility and structure were evaluated in *ttn.2* embryos. Embryos with truncating variants proximal to the *cronos* promoter (e5−/−, e25−/−, e105−/−) had reduced motility compared with sibling control embryos at ≈24 hpf, which was limited to just a tremor when stimulated by touch by 3 dpf (Figure S7A). Immunofluorescence staining for myosin and actin in these 3 dpf mutants showed structured sarcomeres, but some myofibrils were misaligned, and break points were evident in the middle of both slow and fast muscle fibers (Figure 5A; Figure S7B through S7D). MyBP-C and α-actinin appeared normal (Figure S7E and S7F). Immunostaining with T12, recognizing an epitope 3’ of the *ttn.2* truncation site, showed strong staining in both slow and fast muscle fibers, which most likely results from Ttn.1 (Figure S7E; Figure S6N, S6R, S6T, and S6X). Ttn.2 M6 was also detected strongly in e5−/−, e25−/− and e105−/− embryos (Figure S7F through S7H) and is likely to result from Cronos protein. Other M-band proteins localized correctly to the M-band; Myom2 in fast muscle (Figure 5B) and Myom1 in both fast and slow muscle fibers (Figure S7I through S7K). Overall, these data suggest that Ttn.1 and Cronos have a compensatory ruler function and allow many proteins to find their position in the sarcomere in the absence of full-length Ttn.2. However, reduced motility and fiber breakage indicate that sarcomere structure is weaker and prone to damage once contractions commence, possibly due to the absence of Z-disk links by Cronos.

### Severe Loss of Skeletal Muscle Sarcomeric Structure in Embryos With *ttn.2* Truncations Distal to the *cronos* Promoter

The severity of skeletal muscle phenotypes was greater in embryos with truncating variants distal to the *cronos* promoter (e129−/−, 201−/−) compared with those with proximal truncations (e5−/−, e25−/−, e105−/−). The former embryos were barely motile at 24 hpf, showing only weak twitches. By 3 dpf, embryos were paralyzed with no response to mechano-stimulation (Figure S8A). Already at 24 hpf, sarcomeres in e129−/− embryos had immature thin myofibrils resembling early differentiated myofibrils^28^ with only small areas of low-level sarcomeric organization (Figure 5C; Figure S8D). By 2 and 3 dpf, immunofluorescence staining for myosin and actin showed a loss of axial fibrillar organization in both slow and fast fibers (Figure 5A and 5C; Figure S8B and S8C). Western blot analysis shows a significant reduction of slow but not fast MyHC (Figure S9A through S9F). Rather, short lengths of ≈1 µm structures were revealed by staining for α-actinin (Figure 5D) and antibodies that detect Ttn.1, such as T12, Ttn.1 M8-M9, and Z1Z2 (Figure 5E and 5F; Figure S8B). The directionality of these structures was random, differing greatly from normal, organized sarcomeres (Figure 5G and 5H) and resembling I-Z-I complexes. I-Z-I complexes are structures formed during myofibrillogenesis that contain Z-disk components as well as α-actin and titin. These can be put in register on actin filamentous structures and can form in the absence of MyHC.^40^ Ttn.2 (M6 antibody) was not detected in these mutants (Figure 5D; Figure S8E) while other M-band proteins, such as Myom2 were only weakly detected (Figure 5I). This suggests that myofibrils expressing only Ttn.1 have lost their rigidity or their ability to associate side by side, so that they grow in a wavering, random way when viewed longitudinally. Electron microscopy supports this, with a few repeats of sarcomere-like structures running in different directions (Figure 5J through 5M). Between the Z-disks and their associated thin filaments, groups of thick filaments were seen. However, they did not appear to be organized as A-bands, and no M-band structures were seen. Furthermore, the separation of the thick filaments in cross-section was greater than seen in WT siblings. The sarcoplasmic reticulum was present, and some triad structures remain associated with Z-disks (Figure 5L). Overall, the results show that in skeletal muscles of homozygous embryos with *ttn.2* truncations distal to the *cronos* promoter, structures containing I-Z-I brushes and Ttn.1 are formed, but Ttn.1 alone cannot support proper organization of A- and M-bands.

### Sarcomere Structure Disintegrates in Fast Fibers of Embryos With M-Band *ttn.2* Truncation

**Figure 6.**
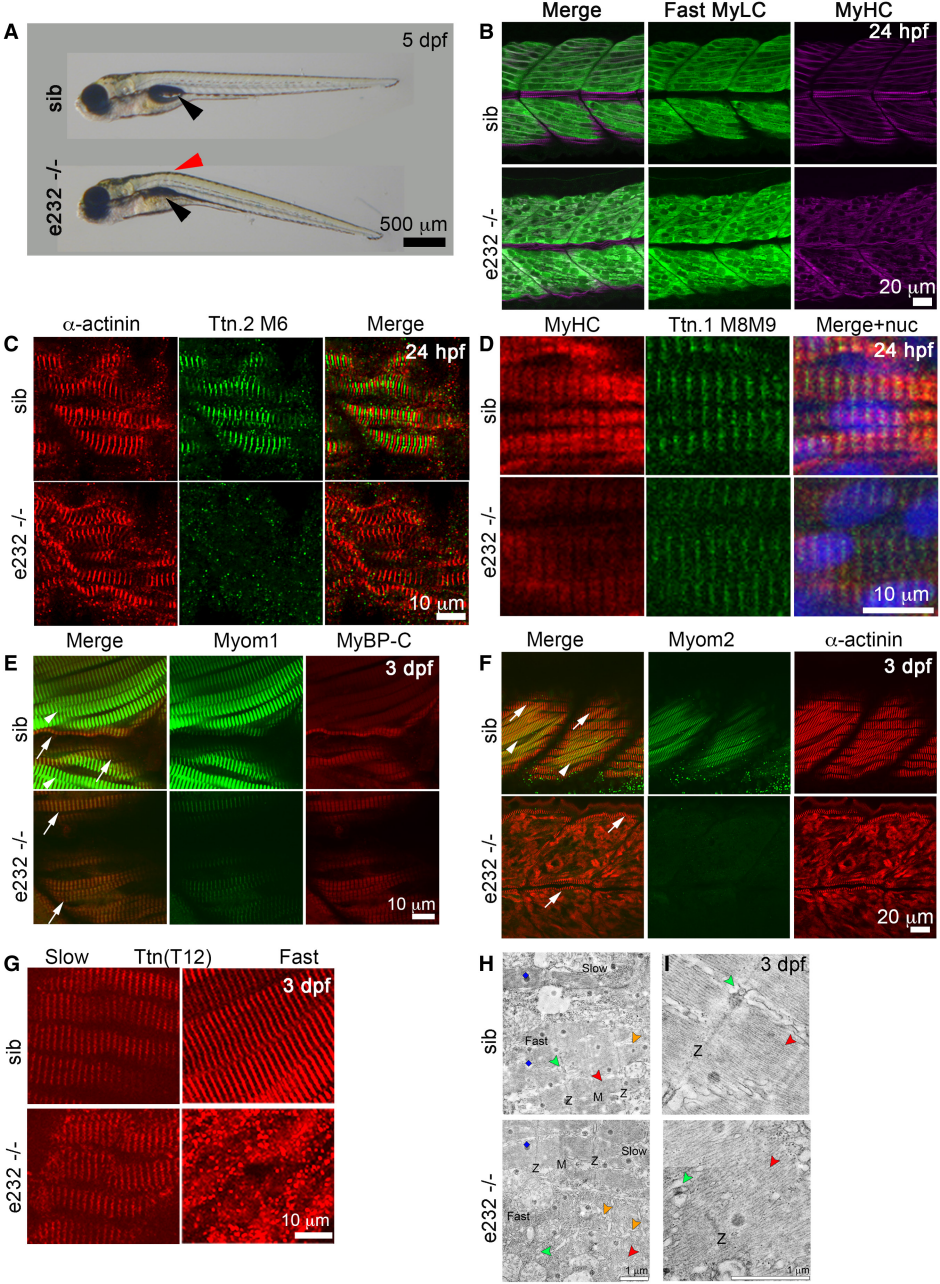
**Skeletal muscle phenotypes in e232−/− embryos. A**, Brightfield images of 5 dpf e232−/− and sibling (sib) control embryos, lateral view, anterior to left. Mutants show a distinctive trunk curvature (red arrowhead) and fail to inflate the swim bladder (black arrowheads). **B** through **G**, Immunofluorescence staining of e232−/− and sibling (sib) control embryos for MyHC (myosin heavy chain), Fast MyLC (fast myosin light chain), α-actinin, Ttn.2 M6, Ttn.1 M8-M9, Myom1 (myomesin-1), MyBP-C (myosin binding protein-C), Myom2 (myomesin-2), and Ttn (titin) T12, shown in lateral view, anterior to left. At 24 hpf (**B**–**D**), MyHC, fast MyLC, α-actinin, and Ttn.1 M8-M9 (slow) show regular sarcomeres, although Ttn.2 M6 is not detected. By 3 dpf (**E**–**H**), Myom1 (strong in fast fibers) and MyBP-C (strong in slow fibers) are present in both slow (**E**, arrows) and fast fibers (**E**, arrowheads) in controls, but are detected only in slow fibers in e232−/− embryos. Myom2 is expressed only in fast fibers (**F**, arrowheads), whereas α-actinin is in both slow (**F**, arrows) and fast (**F**, arrowheads) fibers. In e232−/− embryos, α-actinin is normal in slow fibers (arrows) but is disorganized and aggregated in the deeper fast fibers. Myom2 shows a diminished signal above the background. **G**, Fibers are scanned at different levels to highlight superficial slow muscle (**left**) and deeper medial fast fibers (**right**). Ttn T12 striation patterns are similar in e232−/− and control embryos in slow fibers, but mutants have a punctate, disorganized staining in fast fibers. **H** and **I**, Electron micrographs of longitudinal muscle sections of 3 dpf e232−/− and sibling control embryos. Control embryos have an organized sarcomeric structure with a clear Z-disk (Z) and M-band (M) visible in both slow and fast muscle fibers. e232−/− embryos have organized sarcomeres only in slow muscle fibers. Thick filaments (red arrowheads) are present in mutant fast fibers but are not arranged into sarcomeres. Sarcoplasmic reticulum (orange arrowheads) is disordered in mutant fast fibers. Blue kites indicate EM artifacts. Triads (green arrowheads) are located at the Z-disk in control fast fibers. In mutant fast fibers, there are a small number of triads and Z-discs, but these are not associated together.

Similar to the cardiac findings, the skeletal muscle phenotype in e232−/− embryos was distinct to the other mutants. The overall appearance of e232−/− embryos was indistinguishable from WT siblings at 3 dpf (Figure 3A). However, whereas coiling movement within the chorion was normal at 24 hpf, e232−/− embryos had an abnormal swimming pattern at 48 hpf, which involved slower tail movement and less motion of the trunk. By 5 dpf, e232−/− embryos lacked burst-swimming on touch, unlike WT siblings. However, spasms of the trunk, pectoral fin movement, and increased eye movement in response to touch were detected, indicating intact sensory reflexes (Figure S10A through S10C). Burst-swimming behavior is mediated primarily by fast fibers, while the early coiling movement is mediated by early-differentiating slow muscle.^41,42^ By 5 dpf, e232−/− mutants developed curvature of the trunk and failed to inflate their swim bladders (Figure 6A; Video S1).^42^

At 24 hpf, shortly after differentiation, sarcomeric structure in both slow and fast fibers in e232−/− embryos appeared normal (Figure 6B through 6D). By 3 dpf, however, while slow muscle fibers maintained their regular sarcomeric structure, fast muscle sarcomeres were severely disrupted as indicated by myosin, MyBP-C, α-actinin, and T12 (detecting Ttn.2 upstream from the truncation domain) staining patterns (Figure 5A; Figure 6E through 6G). However, Western blot analysis shows a marked reduction in slow MyHC (Figure S9A through S9F). As in cardiac sarcomeres, Ttn.2 M6 was absent (Figure 6C), but T12 signal was present (Figure 6G), suggesting that truncated Ttn.2 and Cronos were incorporated into the sarcomere. The M-band protein Myom1 is normally present in both slow and fast fibers, but was only detected in e232−/− embryo slow fibers (Figure 6E). Myom2, normally present only in fast fibers, was severely diminished in e232−/− embryos (Figure 6F). Electron microscopy revealed normal sarcomeric structure in e232−/− slow muscle fibers, whereas no sarcomeres were visible in fast muscle fibers, and thick filaments appeared to be highly disordered (Figure 6H and 6I). A small number of Z-discs and triads were also visible, but not associated.

### Adult Cardiac Phenotypes

Heterozygous *ttn.2* mutant fish survived to adulthood with overall normal appearance and swimming behavior. *ttn.2* transcript expression in mutant lines was reduced by ≈50% relative to WT (Figure S11). Confirming previous studies,^14^ neither reduced levels of titin protein nor truncated titin proteins were detectable using Western blots with an N-terminal specific titin antibody, or Coomassie-stained polyacrylamide gels (Figure S12). Due to the lack of an embryonic cardiac phenotype, e232+/− fish were not included in the adult analyses.

**Figure 7.**
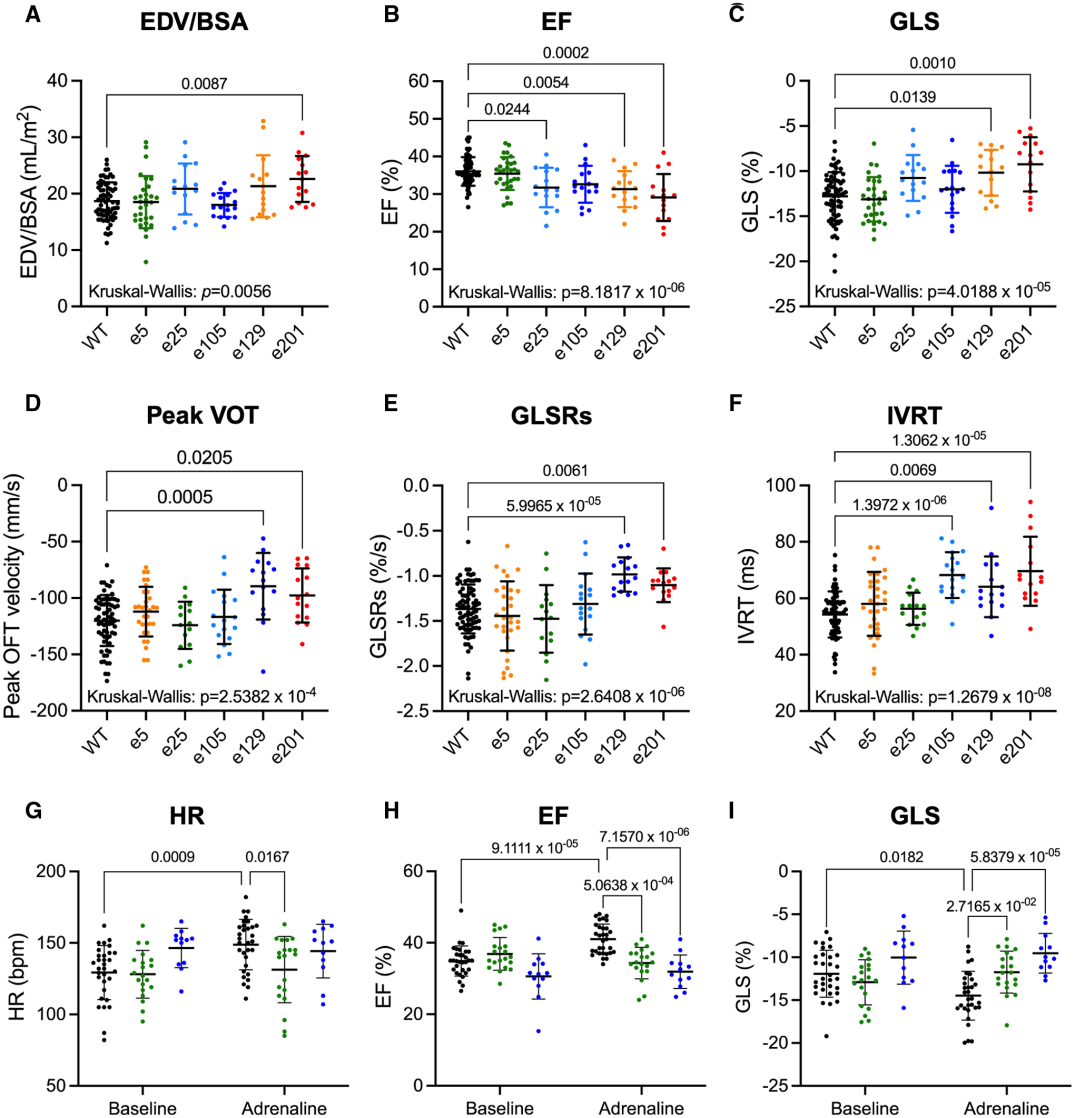
**Ventricular size and function in adult heterozygous *ttn.2* zebrafish.** Cardiac function was assessed in 12-month-old wild type (WT, n=68) and heterozygous (e5, n=30; e25, n=15; e105, n=17; e129, n=15; e201, n=15) *ttn.2* fish using high-frequency echocardiography. **A** through **F**, ventricular function under resting conditions: **A**, indexed ventricular end-diastolic volume (EDV/BSA), **B**, ejection fraction (EF); **C**, global longitudinal strain (GLS), **D**, peak ventricular outflow tract velocity (VOT), **E**, peak systolic global longitudinal strain rate (GLSRs), **F**, isovolumic relaxation time (IVRT). **G** through **I**, heart rate (HR), EF, and GLS responses to acute adrenaline administration (at 2 h) in 10 to 15-month-old WT (n=31), e5+/− (n=20), and e105+/− (n=12) fish. Kruskal-Wallis test with Dunn multiple comparisons (5 comparisons; **A**–**F**), Scheirer-Ray-Hare test with Dunn multiple comparisons (15 comparisons; **G**–**I**), all *P* values adjusted for multiple comparisons using Bonferroni correction, mean±SD, or ∆mean.

Cardiac function in 12-month-old fish was evaluated using high-frequency echocardiography (Figure 7; Table S6). Ventricular end-diastolic volume in the heterozygous *ttn.2* fish was unchanged compared with WT, except for e201+/− that showed modest dilatation (Figure 7A), in line with our previous findings.^13^ The e129+/− and e201+/− lines showed significant impairment of ventricular contractile function, assessed by ejection fraction, global longitudinal strain, and peak outflow tract velocity (Figure 7B through 7D). In addition to systolic impairment, these lines also had prolonged isovolumic relaxation time, indicating diastolic dysfunction (Figure 7F). Ventricular function in the A-band mutant, e201+/−, was similar to the *ttn*^*xu071*^ double mutant (*ttn.2* A-band + *ttn.1* Z-disk truncations),^14^ implicating predominant *ttn.2* effects (Figure S13).

We hypothesized that fish with *ttn.2* truncations outside the A-band might be in a compensated state at baseline, and that exposure to additional stress might unmask latent cardiac dysfunction. We therefore subjected 10 to 15-month-old e5+/− and e105+/− fish to acute adrenaline stress, as previously described.^33^ WT fish demonstrated the expected adrenaline-induced increases in heart rate and contractility (Figure 7G through 7I). In contrast, these responses were not seen in e5+/− and e105+/− fish, indicating a lack of cardiac reserve.

Myocardial structure was further interrogated in e5+/− and e201+/− fish, hypothesizing that these 2 lines are representative of compensated versus penetrant disease states, respectively. No overt differences in wall thickness or trabeculation were observed in hematoxylin and eosin-stained tissue sections in either of these mutant lines relative to WT siblings (Figure S14A). Sarcomere structure, content, and length were also similar to WT (Figure S14B through S14E).

## Discussion

Zebrafish *TTN*tv models exhibit a varying extent of cardiac and skeletal muscle dysfunction that differs with age, variant location, and the expected amount of normally functioning titin protein. Previous zebrafish studies have focused mainly on the developmental impact of titin deficiency using homozygous embryos with selected Z-disk and A-band *ttn.2* truncations.^12^ We now extend these findings by directly comparing *ttn.2* truncations distributed throughout the titin protein, including the first zebrafish model that truncates the M-band. We also provide new data about cardiac phenotypes in a range of adult heterozygous fish. Our data expands understanding of the roles of titin in heart and skeletal muscle and has potential implications for human titinopathies.

Homozygous *ttn.2* embryos showed marked cardiac dysfunction and early death, similar to reported *ttn.2* mutants.^12^ These findings are most readily explained by a lack of functional Ttn.2 protein in the heart and the lack of effective compensation by Ttn.1 or Cronos, despite evidence of some Ttn.1 upregulation. Homozygous *ttn.2* embryos also showed abnormalities of skeletal muscle structure and function, the severity of which appeared to be determined by location with respect to the *cronos* promoter. For proximal truncations, the presence of intact Cronos and Ttn.1 allows formation of fragile sarcomeres that fail following the onset of contraction. However, for distal truncations, both Cronos and Ttn.2 are affected, and although intact, Ttn.1 appears to be insufficient to support viable sarcomere assembly.

The e232−/− embryos were a notable exception with respect to both the cardiac and skeletal muscle phenotypes. A near-complete A-band in e232−/−, capable of interacting with myosin and organizing MyBP-C, seems essential for incorporation and retention of truncated Ttn.2 in sarcomeres. Remarkably, although loss of full-length Ttn.2 protein prevents sarcomere formation, C-terminal titin appears to be dispensable for cardiomyocyte sarcomere assembly, contraction, and normal heart morphology during early development. In contrast to other lines, the minimally truncated Ttn.2 protein is stable enough to allow integration into the sarcomere. Factors such as the truncation position at the distal end of the giant titin protein and the large exon size may result in reduced efficiency of nonsense-mediated decay of e232 alleles.^43^ Although this might be sufficient to support sarcomere development, the presence of truncated titin seems to affect protein interactions in this region. Homozygous mice deficient for titin Mex1 and Mex2 have cardiac lethality through structural changes in the sarcomere that lead to reduced stability and ultimately to disassembly of the sarcomere.^44,45^ When this deletion is made with a late Cre activation, the absence of M-band titin leads to cardiac atrophy.^30^ Of note, sarcomeric integration of C-terminal truncated titin has been reported in human muscle biopsies from patients with M-band *TTN*tv associated with autosomal recessive skeletal±cardiac myopathy.^4–7^

In e232−/− embryos, sarcomere organization was preserved in cardiac and slow muscle, but not in fast muscle fibers. Since *ttn.2* and *ttn.1* are expressed in both slow and fast fibers, these observations suggest that there are fundamental differences in the requirement for C-terminal titin for the assembly and maintenance in these muscle types. One difference between slow and fast skeletal muscle fibers in the M-band region is the expression of myomesin. Whereas Myom1 is present in cardiac, slow, and fast muscle in zebrafish, Myom2 is only expressed in fast skeletal muscle, similar to mammalian muscle (reviewed in Lange et al^39^). In contrast, Myom2 is present in the murine heart from e14.5,^39^ while in fish hearts, expression of *myom2a*, one of the fish *MYOM2* homologs, is detected from the early embryonic heart tube stage. Collectively, our data suggest that Myom2 is not essential for sarcomere assembly but may have a role in the maintenance of sarcomere organization in fast skeletal muscle. Other proteins that interact with M-band titin, such as obscurin, calpain-3, and myospryn, may be important for differentiating normally functioning sarcomeres in fast versus slow and cardiac muscle fibers. Human patients with C-terminal truncations localize MYOM1 and OBSCN (obscurin)/OBSL1 (obscurin-like protein 1) to the M-band, but with some disruptions.^7,46^ Similarly, e232−/− mutants recruited Myom1 to slow and cardiac M-band sarcomeres, possibly via dimerization of Myom1 and binding of Myom1 to obscurin/Obsl1 and myosin. However, the absent M-band/titin connections likely gradually result in structural instability and may underpin the disruption seen in human patient samples.^46^ The selective loss of fast skeletal muscle fibers may reflect susceptibility to damage in response to mechanical and metabolic stress.^47^ Interestingly, dystrophin-deficient mdx mice have disproportionate exercise-induced damage in fast skeletal myofibers, and human patients with Salih myopathy and multi minicore disease associated with C-terminal *TTN*tv show a predominance of slow (type I) fibers from a very early age.^4,5^ Our qPCR data may suggest another possible mechanism for the phenotypic differences between cardiac and fast muscle, differential degradation efficiency of truncated transcripts between muscle and heart in e232−/−, as seen in other tissues.^48^

The location of *TTN*tv has emerged as an important issue in human titinopathies. Congenital skeletal titinopathy is recessive and typically associated with biallelic *TTN*tv, except for a rare dominant *TTN*tv in exon 327.^49^ Cohort studies have demonstrated that these disease-causing *TTN*tv can arise throughout the entire *TTN* gene.^8,50^ Cardiac involvement is seen in ≈50% patients and is more likely when both *TTN*tv are in exons that are constitutively expressed in the adult heart.^8^ Notably, the age of disease onset is much later in patients in whom at least one of the *TTN*tv is present in the last 3 M-band exons.^50^ Only 2 autosomal dominant forms of skeletal myopathy, tibial muscular dystrophy and hereditary myopathy with early respiratory failure, have been associated with single *TTN* missense variants in Fn3-119 (exon344) and Ig-169 in the final M-band exon.^8^ These disorders typically occur in adult life, and cardiac involvement is variable. Taken together, these findings suggest that *TTN* mutation type, location, and titin dose are important determinants of clinical manifestations and disease severity.

The C-terminal part of titin shows unexpected complexity in its integration into the myosin filament, as revealed by recent cryo-electron tomographic structures. Three double strands of titin (dubbed titin alpha and beta) with 6 molecules in total run along the thick filament.^34^ Both titin alpha and beta interact outside the M-band at the first levels of myosin motor domains. However, whereas titin alpha continues into the M-band, the C-terminal regions of titin beta from about the second Ig-domains after the kinase domain could not be traced in the cryo-electron tomographic structures,^34,51^, suggesting that titin beta is either truncated or follows a disordered path. The nonequivalence of 3 of the 6 titin filaments of each half-thick filament may account for the different impact of truncations N-and C-terminal to the kinase domain. Structural studies of the corresponding zebrafish mutants may therefore provide valuable insight into the different pathomechanisms of these mutations.

*TTN*tv are the most common genetic cause of autosomal dominant adult-onset DCM, with the majority of these being in the titin A-band or I/A junction.^2,3^ Interpretation of the clinical significance of *TTN*tv outside this region has remained a clinical conundrum. Our evaluation of adult zebrafish hearts confirms the importance of A-band *TTN*tv, with significant changes in ventricular contraction seen under resting conditions in e129+/− and e201+/− fish. However, e5+/− and e105+/− fish cannot be considered to have normal hearts, as their failure to augment ventricular contraction in response to an adrenaline challenge is indicative of impaired contractile reserve. A recent study found that zebrafish from another *ttn.2* e5+/− mutant had no overt DCM but some mild abnormalities.^52^ All our zebrafish lines had *ttn.2* truncating variants in zebrafish exons that show high sequence homology to constitutively expressed human *TTN* exons. Collectively, our data suggest that *TTN*tv in high PSI cardiac exons confer a genetic susceptibility to DCM and that overt clinical manifestation may depend on patient-related factors such as age, sex, and clinical risk factors, particularly those that increase cardiac mechanical stress. Further human *TTN*tv cohort studies are needed to investigate these points.

Whereas our data, and those from human C-terminal *TTN*tv,^4–7^ show clear evidence for integration of truncated titin protein into the sarcomere, we have no evidence to support such phenomena for more N-terminal truncations. Data for persistent truncated protein obtained from mice and zebrafish have mainly been related to C-terminal A-band truncations.^14,53^ However, recent studies have reported the persistence of some truncated titin protein from I-band and A-band human DCM *TTN*tv cardiomyocytes, but with conflicting evidence regarding its localization in the sarcomere or in intracellular aggregates.^30,54–56^ Factors such as species-specificity, disease type and stage, age, and muscle type may be related to these differences. Our study is not without limitations: while we are confident of the evidence for reduced titin protein expression, titin exon usage in zebrafish postembryonic heart and in postembryonic and adult skeletal muscle remains to be elucidated in detail. Due to potential species differences and the partial yet insufficient compensation by Ttn.1 in zebrafish, our findings may not all be directly translatable to humans. Nevertheless, we provide new insights into the variability underpinning *TTN*tv effects and their potential fundamental impact on thick-filament assembly, and are hypothesis-generating for human clinical studies.

## ARTICLE INFORMATION

### Acknowledgments

The authors thank Dan Hesselson and Xiaolei Xu for assistance with zebrafish lines; Alexander Alexandrovich for assistance with antigen expression and purification; Wolfgang Linke for expert advice on titin protein analysis; the Center for Ultrastructural Imaging, King’s College London, for electron microscopy services; and the Victor Chang Cardiac Research Institute aquarium staff, in particular Aaron Hay and Cecelia Jenkin, for assistance with zebrafish husbandry. The authors acknowledge the Victor Chang Cardiac Research Institute Innovation Center, funded by the NSW Government, including the Preclinical Imaging Facility. The authors acknowledge the support of the King’s College London Poteomics Facility at Denmark Hill. Graphical abstract created in BioRender. Santiago, C. (2026) https://BioRender.com/1u4txan

### Sources of Funding

A.K. O'Brien was supported by the PhD program of the King’s College London British Heart Foundation (BHF) Center of Research Excellence, which also supports M.A.B. Amerudin. M. Gautel holds the BHF Chair of Molecular Cardiology. M. Gautel and Y. Hinits are supported by the European Research Council Synergy Grant 856118, StuDySARCOMERE (Structure and cellular dynamics of the sarcomere). C.F. Santiago was supported by the Simon Lee Foundation and the Australian Department of Education & Training Research Training Program Scholarship. D. Fatkin is supported by NSW Health and the Medical Research Futures Fund.

### Disclosures

None.

### Supplemental Material

Extended Materials and Methods

Tables S1–S6

Figures S1–S14

Video S1

Major Resources Table

Uncropped Western Blots

Proteomics Supplemental Files 2 and 3

References 57–64

## Supplementary Material
