# Supplementary material for: Location-Dependent Differences in Cardiac and Skeletal Muscle Dysfunction Associated With Truncating Titin (ttn.2) Variants
Source: Circ Res. 2026 Jan 7;138(3):e325999. doi: 10.1161/CIRCRESAHA.124.325999 (PMC12854356; doi:10.1161/CIRCRESAHA.124.325999)
Supplement: Supplementary file 2 [file res-138-e325999-s002.pdf]

Location-Dependent Differences in Cardiac and Skeletal Muscle Dysfunction Associated With Truncating Titin (*ttn.2*) Variants

SUPPLEMENTARY MATERIAL

TABLE OF CONTENTS

Detailed Methods		2
Supplemental Figures		
Figure S1	Generation of zebrafish *ttn.2* truncation variants.	13
Figure S2	Differences between the Ttn.2 and Ttn.1 M-band regions and specific antibodies.	14
Figure S3	Cardiac function in heterozygous *ttn.2* mutant embryos.	15
Figure S4	Abnormal heart development in *ttn.2* mutants.	16
Figure S5	Zebrafish *ttn.1* truncation variant.	18
Figure S6	Expression of titin in embryonic somitic muscle and heart.	19
Figure S7	Homozygous embryos with *ttn.2* tv proximal to the *cronos* promoter have abnormalities in muscle fiber structure and motility.	21
Figure S8	Homozygous embryos with *ttn.2* tv distal to the *cronos* promoter have severe abnormalities in muscle structure and function.	23
Figure S9	Evaluation of MyHC levels by western blot.	24
Figure S10	*ttn.2* e232-/- embryos maintain motility of various muscles.	25
Figure S11	*ttn.2* transcript expression in adult heart	26
Figure S12	Evaluation of titin levels in adult zebrafish heart.	27
Figure S13	Comparison of ventricular size and function in adult heterozygous *ttn.2* and *ttn.2*/*ttn.1* double mutant zebrafish.	28
Figure S14	Normal cardiac morphology and sarcomeric structure in adult heterozygous *ttn.2* zebrafish.	29
Supplemental Tables		
Table S1	Primers used for genotyping of mutant zebrafish lines.	30
Table S2	Primers used for qPCR analysis.	31
Table S3	Primers used for in situ hybridization (ISH) probes.	33
Table S4	Antigen sequences for antibody production.	34
Table S5	Zebrafish *ttn.2* exons targeted in the mutant lines evaluated and corresponding human *TTN* exons.	35
Table S6	Echocardiographic assessment of adult *ttn.2* zebrafish.	36

Materials and Methods

Please see the Major Resources Table in the Supplemental Materials.

Zebrafish husbandry

Zebrafish (Danio rerio) were raised at a density of 10-15 fish per 3L tank and were maintained as per standard procedures [18]. All experiments using zebrafish were performed in compliance with relevant laws and institutional guidelines in accordance with: (i) protocols approved by the Garvan Institute of Medical Research/St Vincent's Hospital Animal Ethics Committee and the Institutional Biosafety Committee (Australia) and (ii) licenses held under the UK Animals (Scientific Procedures) Act 1986 and later modifications (United Kingdom).

Generation of zebrafish lines

Zebrafish lines carrying 6 truncating mutations in different regions of the *ttn.2* gene were generated (Figure S1). Exon numbers are based on [11]. The *ttn.2*^{e5} line (e5; Figures 1A and S1A) was generated on the AB background using ENU mutagenesis, followed by genome sequencing mapping [57-60]. *ttn.2*^{e25} (e25; Figures 1A and S1B), *ttn.2*^{e129} (e129; Figures 1A and S1D) and *ttn.2* ^{e232} (e232; Figures 1A and S1F) lines were generated using clustered regularly interspaced short palindromic repeats (CRISPR) - CRISPR associated protein 9 (Cas9) - mediated gene editing. The e25, e129, and e232 lines were maintained on a Tupfel Long Fin (TupLF) background. The *ttn.2*^{e105} line (e105; Figures 1A and S1C) was generated on the TE background using transcription activator-like effector nuclease (TALEN)-mediated genetic engineering. The *ttn.2*^{e201} line (e201; Figures 1A and S1E), has previously been described [13]. The e5, e105, and e201 lines were maintained on a TE background. Variants were confirmed by Sanger sequencing. Sequencing of *ttn.1* at the orthologous locus showed unaltered *ttn.1* sequence in the *ttn.2* mutants.

The *ttn.1* e7 line (Figure S2) was obtained by injecting zygotes with EnGene Spy Cas9 NLS protein (New England Biolabs, Ipswich, Massachusetts, USA) with Alt-R CRISPR-Cas9 crRNA and tracrRNA (Integrated DNA Technologies, Inc., Coralville Iowa, USA) to target GCTTGCGTTGACATGATAGA**AGG** in the Z-disk region of *ttn.1*. The *ttn*^{xu071} line (kindly provided by Xiaolei Xu) has a double truncation (*ttn.2* A-band and *ttn.1* Z-disk) and has been described in detail [14]. Genotyping of all lines was performed by polymerase chain reaction (PCR) amplification of genomic DNA extracted from whole embryos or adult fin clips, followed by Sanger sequencing or restriction enzyme digest, using the primers and enzymes listed in Table S1.

qRT-PCR

Total RNA from pooled wild-type (WT), as well as homozygous and heterozygous *ttn.2* embryos (30 embryos/sample, n=5 samples, 3-5 dpf), or from pooled heart-enriched WT and homozygous *ttn.2* embryos (made by dissecting out most of the somitic and craniofacial muscle by 2 cuts under dissecting microscope, 25 embryos/sample, n=5 samples, 3-5 dpf), or from pooled WT and heterozygous *ttn.2* adult hearts (2 hearts/sample, n=5 samples) was extracted using TRIzol (Sigma-Aldrich, St. Louis, Missouri, USA) and the RNeasy Micro Kit (Qiagen, Hilden, Germany) as described [19]. Purified RNA (1000ng) was used to generate cDNA using the Superscript III First-Strand Synthesis System (Invitrogen, Burlingame, California, USA). Relative qPCR was carried out in 384-well plates using a Light Cycler 480 thermal cycler (Roche, Basel, Switzerland) with primers listed in Table S2. Primer efficiency for qPCR was tested using serial dilution of cDNA and generation of standard curves. Gene expression was normalized to the expression level of the housekeeping genes *tmem50a* and *ube2a* using ΔΔCt (cycle threshold) values and graphed relative to transcript expression in WT fish or as absolute expression.

***In situ* mRNA hybridization, and immunofluorescence**

In situ mRNA hybridization was performed as described [20]. Probes used were kinase, N2A, and N2B for *ttn.2* and *ttn.1,* and Cronos [16] (primers listed in Table S3). For immunohistochemistry, primary antibodies were: α-actinin, 1:500 (A7811; Sigma-Aldrich), actin (Actc1a), 1:100 (GeneTex, Irvine, California, USA), myosin binding protein C (MyBP-C), 1:200 [21], an antibody made against human cardiac C0-C1 that recognises cardiac, slow (strongly) and fast (weakly) skeletal isoforms in zebrafish, Myom1 (myomesin B4, 1:100 [22]), Myom2 (M-protein, AA259) [23], sarcomeric myosin heavy chain (MyHC; A4.1025), 1:10 [24], fast myosin light chain (fast MyLC, F310), 1:10 [25], titin (T12), 1:10 [26], titin (Z1Z2), 1:100 [27], Ttn.2 (Ttn.2 M6, see below), 1:1000, Ttn.1 (Ttn.1 M8-M9, see below), 1:2000. Secondary antibodies were mainly Alexa dye-conjugated (Thermo Fisher Scientific, Waltham, Massachusetts, USA), as well as Goat anti IgA FITC (F9384, Sigma-Aldrich) and Cy3 AffiniPure Goat Anti Mouse IgG, Fcγ fragment specific (Jackson ImmunoResearch Laboratories Inc., West Grove, Pennsylvania, USA). Samples for immunohistochemistry were fixed and stained as previously described [28] and imaged on a Zeiss LSM510. Samples were mounted in agarose and photographed on a Zeiss Axiophot with Axiocam (Carl Zeiss, Oberkochen, Germany) using Zen software (Carl Zeiss). For all confocal imaging of immunofluorescence or for in situ hybridisation, groups of 25-40 embryos from WT or incrosses of identified carriers were used for each analysis. Assessment of phenotype was done blindly and at least 5-7 selected embryos from each phenotype/identifiable level of staining were imaged and subsequently genotyped by PCR method as detailed for each mutant line in Supplementary Table 1. Images most suitable to represent each group were used for IF and in situ hybridisation figures. For new antibodies produced for this work (see below) or that were used for the first time in zebrafish, initial testing at various fixation regimes and antibody

dilutions were performed on WT embryos with controls such as secondary antibody only controls and already tested counter staining to define specificity such as correct sarcomeric binding.

Antibody production and testing

To generate specific antibodies to M-band zebrafish Ttn.1 and Ttn.2, we identified unique regions in M-band Ttn.1 and Ttn.2 chosen from sequence alignments (Table S4). The M6 Ttn.2 antibody was designed for a unique region that shares high homology to human M6 but is missing from Ttn.1. The M8-M9 Ttn.1 antibody was designed for a region homologous to the human Mis6-M8-M9-Mis7 region in Ttn.1. The cDNA constructs for protein expression were amplified from embryonic zebrafish cDNA and primer design was based on *ttn.2* and *ttn.1* sequences (GenBank accession no. DQ649453.1). *ttn.2* or *ttn.1* DNA was cloned into a modified pET vector with an N-terminal His_6-tag and TEV cleavage site. The identity of the derived constructs was verified by DNA sequencing. Ttn.2 is4-M6 and Ttn.1 is6-M8-M9-is7 fragments were expressed in the *E. coli* strain BL21-CodonPlus (DE3)-RIPL (Agilent Technologies, Santa Clara, California, USA). Protein was expressed in auto-induction media containing lactose to drive expression of the T7 promoter and cells were harvested by centrifugation and lysed using B-PER Bacterial Protein Extraction Reagent (Thermo Fisher Scientific). Protein was purified by nickel-affinity purification and gel filtration chromatography. After TEV protease cleavage of the His6-tag, Ttn.2 is4-M6/Ttn.1 is6-M8-M9-is7 fragment were used for immunization of rabbits and polyclonal sera were collected (Eurogentec, Seraing, Belgium). The antigen was coupled to NHS-activated Sepharose 4 Fast Flow beads (GE Healthcare, Chicago, Illinois, USA) and serum was affinity-purified as previously described [61].

Titin protein gels & Western blots

Zebrafish embryo tails (n = 20 tails per biological replicate) were homogenized in thiourea sample buffer (8 M urea, 2 M thiourea, 75 mM DTT, 50 mM Tris-HCL pH 6.8, 3% (w/v) SDS, 10% (v/v) glycerol, 0.05% (w/v) bromophenol blue) supplemented with protease inhibitors. Solubilised samples were centrifuged at 12,500 RPM for 2 minutes, snap-frozen in liquid N_2 and stored at -80ºC or heated at 40ºC for 20 minutes for immediate loading.

Titin isoforms were resolved in 1% SDS-SeaKem Gold agarose (Lonza) gels using a modified protocol, performed at 7.5 mA/gel [29]. Total protein levels were visualised on a ChemiDoc MP system (Bio-Rad) by addition of 2,2,2-trichloroethanol (ThermoFisher Scientific, 139441000) to the gel prior to polymerisation. ImageLab (Bio-Rad) software was used to analyse the relative optical density of titin bands, which were calculated from n = 3 gels per experiment. For Western-blot detection of N- and C-terminal titin, proteins were transferred to a nitrocellulose membrane (Amersham Biosciences) and probed with anti-titin antibodies Z1Z2 (1:1000) [8] and Ttn.2 M6 (1:1000), respectively. To analyse MHC isoforms, samples were separated on standard 10% SDS-PAGE gels, then transferred for Western-blot analysis. Ponceau S was used to determine transfer efficiency, and densitometry was performed using the ImageLab software. Primary antibodies used for WB were: DA-D5 (slow MyHC, DSHB) [62], S58 (slow MyHC, DSHB) [63], EB165 (fast MyHC, DSHB) [64], GAPDH (Proteintech). Secondary antibodies used for WB were: polyclonal goat anti-mouse IgG/HRP (Agilent P0447), goat anti-rabbit IgG (H+L)/HRP (Merck Millipore 401315) and IRDye® 800CW donkey anti-mouse (LI-CORbio 925-32212).

Hearts (ventricle and atrium only) from adult WT and heterozygous *ttn.2* zebrafish (male, ~14 months old) were snap frozen in liquid nitrogen (n=2 per sample). Hearts were homogenized on ice in a urea buffer as previously described [30]. Samples were loaded onto a 2.0% agarose-stabilized polyacrylamide gel at a concentration of 15-20 μg under denatured and reduced

conditions. Following SDS- polyacrylamide gel electrophoresis (PAGE), gels were either stained immediately using Coomassie blue to visualize total protein or transferred onto 0.2 µm PVDF membranes which were then immunostained for titin. Antibodies used: anti-*TTN* mouse monoclonal antibody (2F12, 1:1000, Abnova, Taipei, Taiwan, #H00007273-M07A), anti-mouse HRP (1:15000, Cytiva, Marlborough, Massachusetts, USA, #NA9310).

Proteomics Analysis

Whole zebrafish 3dpf embryos lysates were lysed within 6M urea buffer containing 5.4% SDS, 4.45% b-mercaptoethanol, 2.3% NP-40, 136.4 mM Tris (pH 6.8) with proteinase inhibitor and phosSTOP. Lysates were aliquoted and precipitated using cold acetone. The protein pellets were resuspended in 100mM triethylammonium bicarbonate buffer (TEAB), tryptic digested at a ratio of 100:1 (protein : trypsin) at 37°C overnight, after being treated with Dithiothreitol (DTT) regent for reduction followed by iodoacetamide (IAA) alkylation. The digested peptides were desalted on C18 spin column and dried by SpeedVac prior to LCMS analysis. The tryptic peptides were directly ionized within the Easy-spray ion source (Thermo) and injected into Orbitrap Eclipse Tribrid mass spectrometry (Thermo Fisher Scientific) coupled with Ultimate 3000 RSLC nano system for analysis. For liquid chromatography, a reverse phase Thermo Acclaim Pepmap trap column (2cm length, 75µm in diameter and 3µm C18 beads) were connected to the nanoflow HPLC on an Easy-spray C18 nano column (50 cm length, 75 µm in diameter, ThermoFisherScientific). Buffer A (5% ACN, 0.1% formic acid) and buffer B (80% ACN, 0.1% formic acid) were used and peptides were eluted in standard gradient procedure.
The MS instrument was operated in the positive ion mode with an electrospray through a heated ion transfer tube at 275C. MS DIA datasets were acquired within Xcalibur 4.7 using the following parameters: scan range 400-900m/z, MS resolution of 60,000 at m/z 200, a

normalized AGC target (%) 250, and maximum injection time of Auto. The MS/MS scan was performed in HCD mode. All data were acquired in positive polarity and centroid mode.

Resulting DIA raw files were searched against the Uniprot/TrEMBL database (Zebrafish) following analysis pipeline within PEAKS Studio software (Bioinformatics Solutions Inc, version 12). DIA DB search parameters: precursor and fragment mass error tolerances (auto detected) with match between run, trypsin as enzyme with 1 miss cleavage, cysteine carbamidomethylation as a fixed modification, peptide filter FDR 1%. Label-free quantification was applied here with DIA LFQ workflow embedded, using high precision mode, default filter setting for peptide and protein and TIC normalization. The extracted precursor ion intensities were applied for downstream analysis. Two-sample t-test algorithm was applied for the differential analysis.

The mass spectrometry proteomics data have been deposited to the ProteomeXchange Consortium via the Proteomics Identification Database (PRIDE) [31] partner repository with the project accession number: PXD071430.

Electron microscopy

Muscle contraction was inhibited using 20 mM 2,3-butanedione monoxime and embryos were fixed in a solution of 4% paraformaldehyde/2.5% glutaraldehyde in PBS for 1 h on ice, post-fixed in a 1% osmium tetroxide in PBS solution for 30 m on ice, followed by graded dehydration in ethanol on ice. Sections were stained with uranyl acetate or UranyLess (Electron Microscopy Services, Hatfield, Pennsylvania, USA). Electron microscopy was carried out using a JEOL JM1400 transmission electron microscope in the Centre for Ultrastructural Imaging, King's College London.

Video-microscopy

Heart rates were obtained by manually counting the number of beats that occurred during 15 s of each video and multiplying this by 4 to obtain the number of beats per minute. End-diastolic (EDA) and end-systolic (ESA) chamber areas were derived by measuring the short (a) and long axis (b) diameters of each chamber in video frames frozen during diastole or systole using the NIS Elements software line tool. Chamber area was then calculated according to the formula: $A = \pi * ½ a * ½ b$. Fractional area change (FAC) was derived using the formula: FAC = (EDA-ESA)/EDA. [13] Measurements were averaged from at least two different cardiac cycle from within the same video.

High frequency echocardiography

Underwater echocardiography was performed in adult zebrafish aged 9-15 months using the Vevo3100® Imaging Station (VisualSonics, Amsterdam, Netherlands) equipped with a high frequency transducer (MS700D) as described [13,32]. Male fish were used in this study. We have previously reported that there is considerable variability in echocardiographic measurements in female fish due to technical issues, e.g. distortion of the heart's position due to eggs, and varying body weight depending on the number of eggs and gravity status. The latter confounds use of body weight for chamber size normalization [32]. Two-dimensional (B-Mode) images, color and pulsed-wave Doppler signals were recorded in the long axis view optimized for either ventricular or atrial assessment, respectively. Image analysis was performed using the VevoLab™ analysis software package version 5.7.0 (VisualSonics) [13,32] by a single operator who was blinded to genotype. B-Mode images in the long axis view were used to derive ventricular end-diastolic and end-systolic volumes (EDV, ESV), and maximal atrial size (atrial area, AA), indexed to body surface area (BSA). Speckle tracking analysis of ventricular wall motion was performed with the VevoStrainTM analysis software package (VisualSonics) and used to calculate heart rate, ejection fraction (EF), global longitudinal strain (GLS) and global

longitudinal strain rate (GLSR). Pulsed-wave Doppler signals measured include: ventricular outflow tract velocity (VOT), E wave (peak velocity of blood inflow across the atrioventricular valve during early diastole), A wave = peak velocity of blood inflow across the atrioventricular valve during atrial systole, and isovolumic relaxation time (IVRT).

Adrenaline Stress

10-15 month old heterozygous e5 (e5+/-) and e105 (e105+/-) mutants and WT siblings were subjected to acute adrenaline stress by submersion in 500 µM epinephrine hydrochloride (E4642, Sigma-Aldrich) for 2h [33]. Systolic and diastolic function were measured at baseline and at the conclusion of adrenaline exposure using high-frequency ultrasound.

Statistical analysis

Statistical analyses were performed with GraphPad Prism (GraphPad Software, Inc., California, USA) unless otherwise specified. Normality testing was performed using the Shapiro-Wilk method. For data that were not normally distributed or for sample sizes that were small (<10/group), comparisons between groups were made using non-parametric tests such as Kruskal-Wallis test (instead of ordinary one-way ANOVA) or Scheirer Ray Hare analysis (instead of ordinary two-way ANOVA). Scheirer Ray Hare analyses were performed using RStudio. Where significant factor or interaction effects were found, data with multiple groups was further tested using either Dunn's or Tukey's multiple comparisons tests. All p-values derived from these tests were adjusted for multiple comparisons using Bonferroni's correction. Where p-values were $p<0.0001$, data was re-analysed using the same statistical tests using RStudio to obtain exact p-values. All p-values are exact unless otherwise stated.

Data points in plots represent biological replicates, with bars representing mean ± SD unless otherwise specified. The absolute difference between two groups is reported in the text as the delta (Δ) of the mean ± SD (Δmean ± SD). Significance level (α) was $p \leq 0.05$ for all studies.

Randomization and Blinding Procedures

Experiments involving mutant and WT sibling zebrafish embryos were randomized as follows: Immunofluorescence and *in situ* hybridisation were done in tubes containing embryos from a cross of heterozygote parents used for the experiment containing all unknown genotypes, imaged, analyzed and then subsequently genotyped. Embryonic heart physiology was also done by video imaging embryos from a heterozygote in-cross, analyzing videos and genotyping afterwards. However, blinding was not performed in EM experiments due to the small number tested. Also, in qPCR embryos or adults were genotyped first before being pooled in groups for experiments.

Power calculations for adult zebrafish echocardiography were based on previously published calculations [32]. Operators were not blinded to genotype during echocardiography acquisition for any experiment; however, animals from within genotypes were randomly selected to be included in the study from a larger pool of siblings. Echocardiography data were randomized by assigning each fish a random number and then blinded for genotype and/or treatment by a person other than the analyzer.

For analysis of adult heart tissue sections, whole heart images from all three zebrafish lines were acquired unblinded. All images were collated into a single folder and blinded by a person other than the analyzer, who assigned each image name a coded ID. Following analysis of all images, the analyzer was then unblinded to the genotype of each image in order to plot the data. Representative images were selected based on: (1) the acquisition of the image in the 'valvular plane' (both the atrio-ventricular and bulbo-ventricular valve present) to improve

standardization across images, and (2) whether the appearance of the image aligned with the quantified data. Additionally, care was taken to note the presence/absence of any distinct changes in tissue morphology in images of hearts from multiple animals which could represent a phenotype before representative images were selected. During analysis of individual sarcomeres, images were batch analyzed using a custom written ImageJ macro in which all images were analyzed using the same image segmentation threshold.

Author contributions:

Celine F. Santiago- study design, data acquisition, data analysis, co-wrote original MS, review of final MS. Inken G Huttner- study design, data acquisition, data analysis, co-wrote original MS, review of final MS. Ailbhe O Brien- study design, data acquisition, data analysis, co-wrote original MS, Pauline M. Bennett- data acquisition, data analysis. Melissa A. B. Amerudin- data acquisition, data analysis. Jasmina Cvetkovska- data acquisition, data analysis, review of final MS. Renee Chand - data acquisition, data analysis, review of final MS. Mark Holt- data analysis. Gunjan Trivedi- data acquisition, data analysis, review of final MS. Louis W. Wang- data acquisition, data analysis, review of final MS. Xiaoping Yang- Proteomics data acquisition, data analysis. Kelly A. Smith- generation and mapping of ZF ENU mutant, review of final MS. Mathias Gautel- study design, data analysis, supervision, funding, co-wrote final MS. Diane Fatkin- study design, data analysis, supervision, funding, co-wrote final MS. Yaniv Hinits- generation of CRISPR/Cas9 mutants, study design, data analysis, supervision, co-wrote original and final MS.

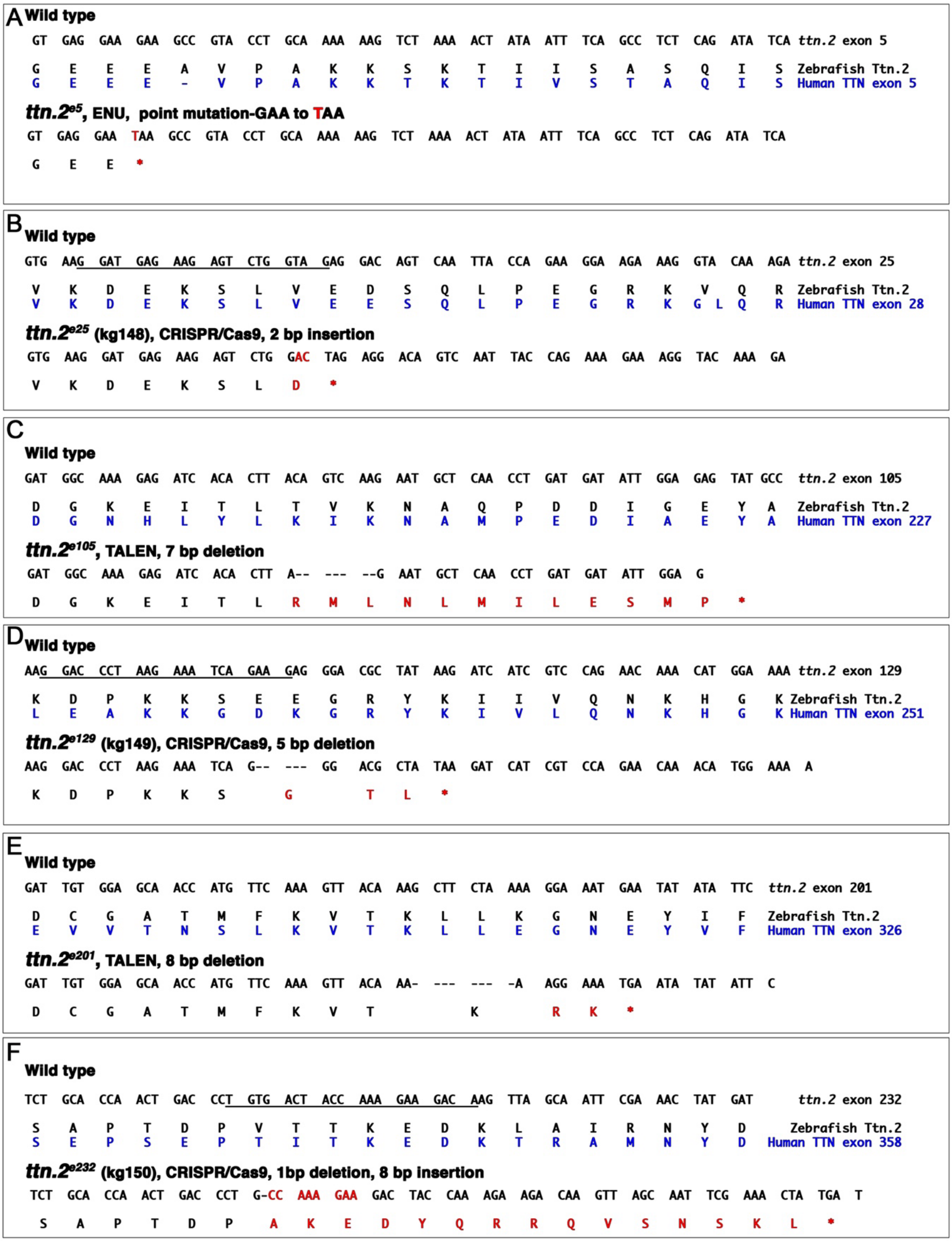

Figure S1. Generation of zebrafish *ttn.2* truncation variants. A through **F,** Sequence information and methods used to generate *ttn.2* truncation alleles. Rows show DNA and protein sequences for wild-type and *ttn.2* mutant zebrafish (black) and corresponding human protein sequence (blue). CRISPR target sequences are underscored. In the mutant allele sequences, deleted bases (dashed lines) and insertions (red) are shown. Resulting amino acid changes are shown in red. Exon numbering is based on Seeley et al ([11]) for zebrafish and the inferred complete human *TTN* meta-transcript (NM_001267550.2).

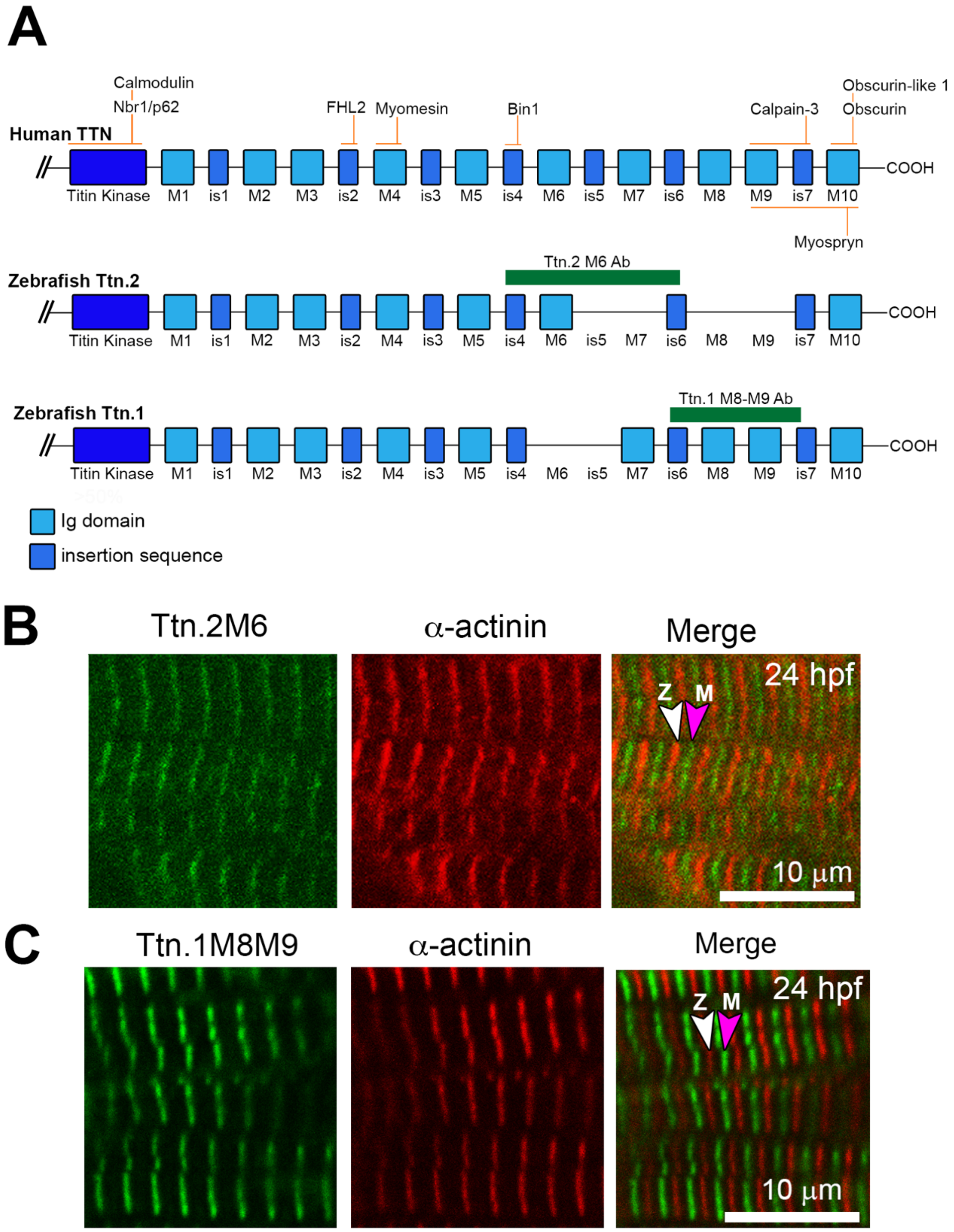

Figure S2. Differences between the Ttn.2 and Ttn.1 M-band regions and specific antibodies. A, Comparison of M-band protein domains in human titin and zebrafish Ttn.2 and Ttn.1. Immunoglobulin-like domains M1 to M10 (light blue), unique insertion sequences 1 to 7 (is; dark blue) and antibody epitopes made in this study (green bars) are shown. Known sites of interacting proteins are denoted above. **B** and **C**, Immunofluorescence staining of somites from wild-type embryos at 24 hpf using Ttn.2 M6, Ttn.1 M8-M9, and α-actinin. Both Ttn.2 M6 and Ttn.1 M8-M9 detect Ttn protein localized to the M-band (M) in sarcomeres alternating with α-actinin in the Z-disk (Z).

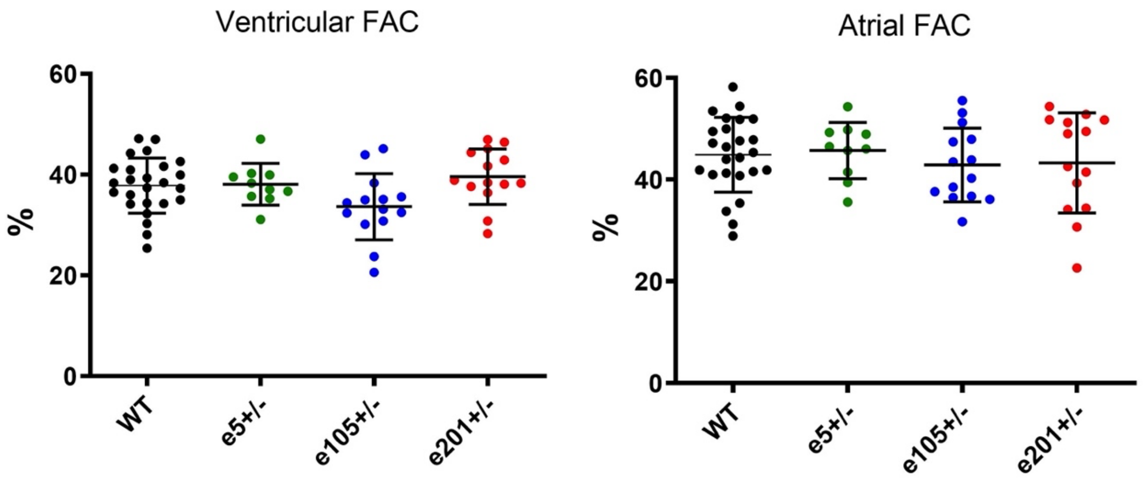

Figure S3. Cardiac function in heterozygous *ttn.2* embryos. Ventricular and atrial contractile function were assessed in 3 dpf wild-type (WT) and heterozygous mutant (e5+/-, e105+/-, e201+/-) embryos using video-microscopy. Data are expressed as fractional area change (FAC; %). Unpaired t-tests, mean ± SD. One-way ANOVA, mean ± SD, WT n=25, e5+/- n=10, e105+/- n=14, e201+/- n=14.

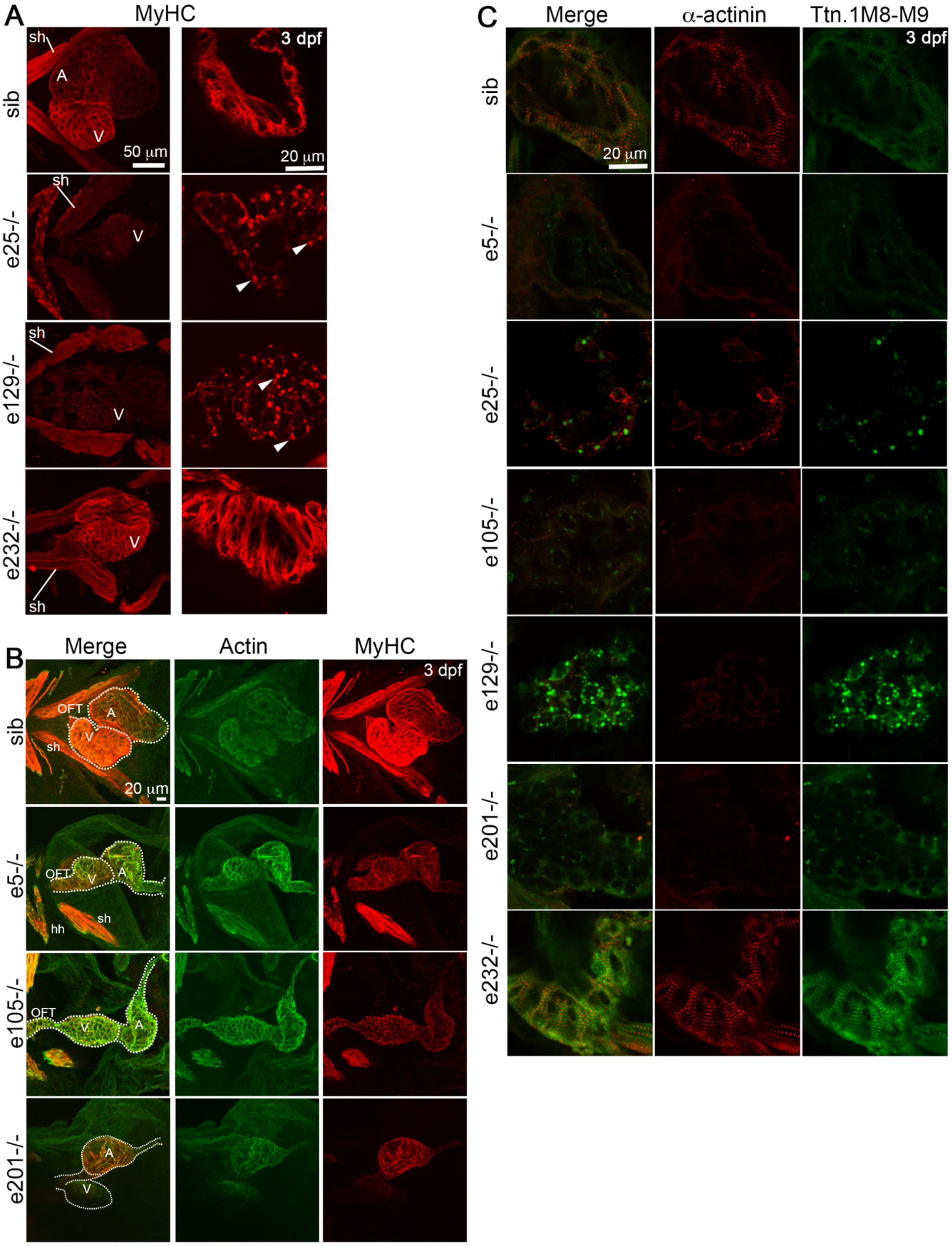

Figure S4. Abnormal heart development in *ttn.2* mutants. A, Confocal stacks (left panel) or single scan higher magnification (right panel) of hearts from 3 dpf e25-/-, e129-/- and e232-/- embryos and a sibling (sib) control immunostained for myosin heavy chain (MyHC) shown in ventral view, anterior to left. Heart chambers in e25-/- and e129-/- embryos are small and linear, and the sternohyoideus muscle is pushed outwards by edema. **B,** Confocal stacks of hearts from 3 dpf e5-/-, e105-/- and e201-/- embryos immunostained for actin and MyHC shown

in ventral view, anterior to top. Heart chambers in these mutants are also small and linear. **C,** Single confocal high magnification scans of 3 dpf ventricular cardiomyocytes of homozygous ttn.2 and sibling control embryos, immunostained for α-actinin and Ttn.1 M8-M9 shown in ventral view, anterior to left. Cardiomyocytes show very low levels of α-actinin and Ttn.1 M8-M9 in all mutants but myocardial morphology and sarcomeres in e232-/- embryos are similar to control. A denotes atrium, hh, hyohyoideus, OFT, outflow tract, sh, sternohyoideus, and V, ventricle.

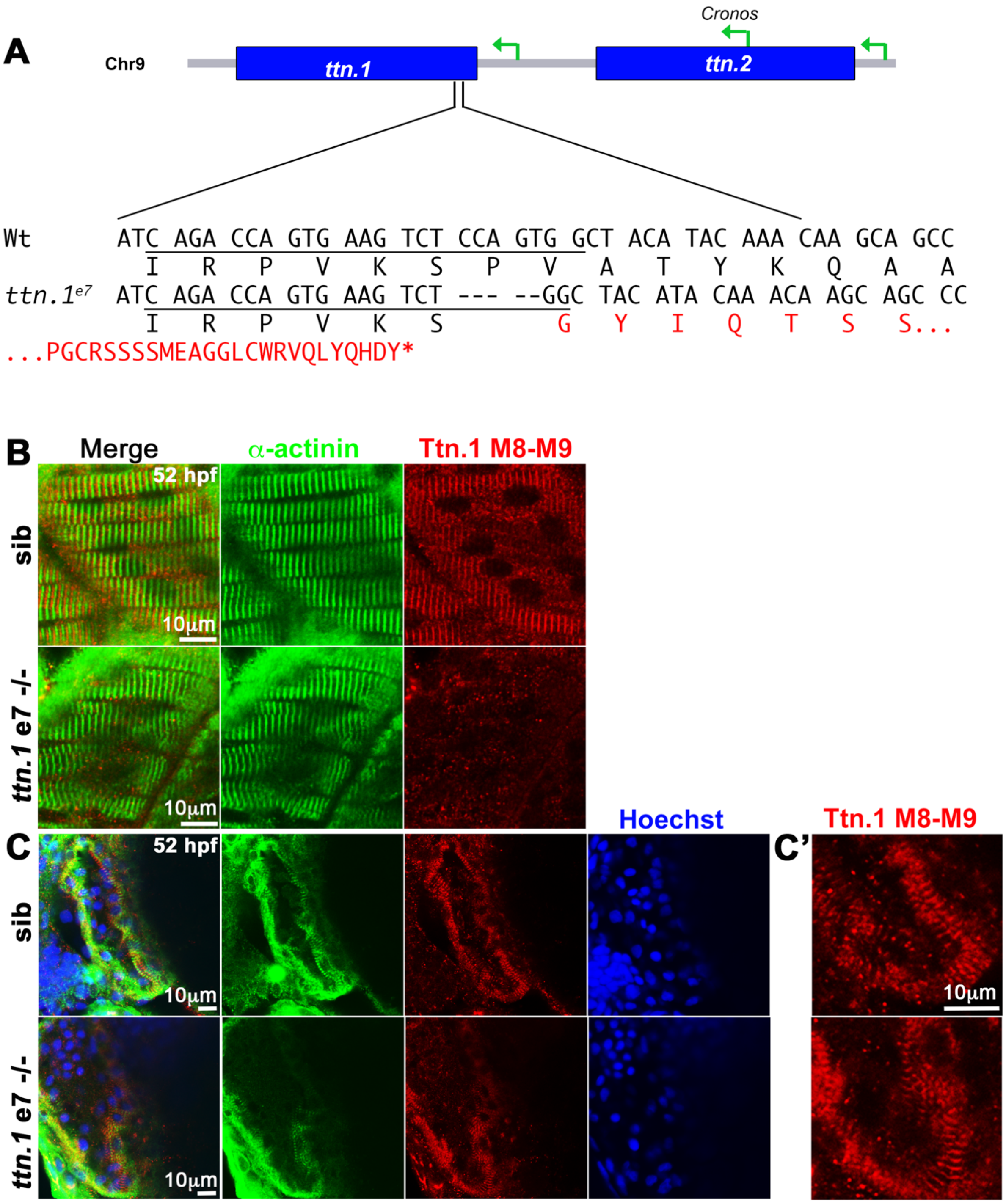

Figure S5. Zebrafish *ttn.1* truncation variant. **A**, DNA and protein sequence for wild-type (WT) and *ttn.1* e7 alleles. CRISPR target sequences are underscored. In the mutant sequences, deleted bases (dashed lines) and altered amino acids (red) are shown. **B** and **C**, Immunofluorescence staining of somitic slow muscle and heart in 52 hpf *ttn.1* e7-/- fish and a genotyped WT sibling for α-actinin and Ttn.1 M8-M9, shown in lateral view anterior to left. Ttn.1 M8-M9 signal is strongly reduced in *ttn.1* e7-/- somitic slow muscle fibers (**B**) but remains strong in cardiomyocytes (**C** and **C'**, higher magnification) indicating that it can also detect Ttn.2.

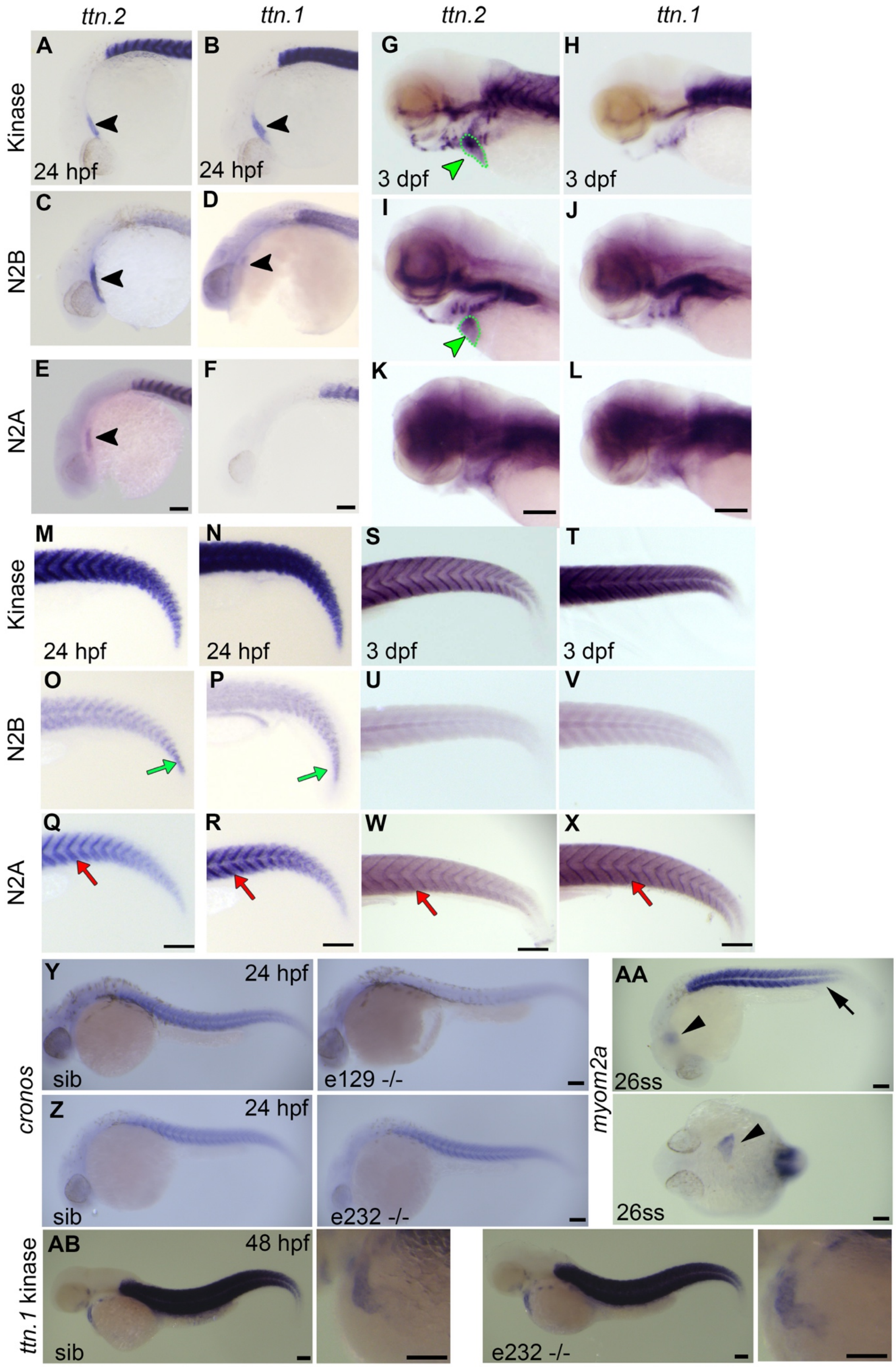
ttn.2
ttn.1
ttn.2
ttn.1
Kinase
N2B
N2A
A
B
G
H
24 hpf
24 hpf
3 dpf
3 dpf
C
D
I
J
E
F
K
L
Kinase
N2B
N2A
M
N
S
T
24 hpf
24 hpf
3 dpf
3 dpf
O
P
U
V
Q
R
W
X
cronos
Y
24 hpf
sib
e129 -/-
Z
24 hpf
sib
e232 -/-
myom2a
AA
26ss
26ss
ttn.1 kinase
AB
48 hpf
sib
e232 -/-

Figure S6. Expression of titin in embryonic somitic muscle and heart. Whole mount in situ hybridization using riboprobes for kinase, N2B and N2A of *ttn.2* and *ttn.1* (purple) in wild-type embryos at 24 hpf (**A-F**, **M-R**) or 3 dpf (**G-L**, **S-X**), lateral view, anterior to left. At 24 hpf, both *ttn.2* and *ttn.1* show expression (black arrowheads) with kinase and the N2B probes (**A**,**C** and **B**,**D**, respectively) but not with *ttn.1* N2A (**E**,**F**). By 3 dpf, expression (green arrowheads) in the heart (green dotted outline) is only detected with *ttn.2* kinase (**G**) and *ttn.2* N2B probes (**I**) but not with *ttn.2* N2A (**K**) or any of the *ttn.1* probes (**H**,**J**,**L**). At 24 hpf, *ttn.2* kinase and *ttn.1* kinase are strongly expressed in somites (**M**,**N**); *ttn.2* and *ttn.1* N2B are diffuse throughout somites (**O**,**P**) with higher levels in the most caudal somites (green arrows); *ttn.2* and *ttn.1* N2A are diffusely present (**Q**,**R**) and concentrated along somite borders (red arrows). At 3 dpf, both *ttn.2* and *ttn.1* kinase are present throughout the somites (**S**,**T**); *ttn.2* N2B and *ttn.1* N2B are only weakly expressed (**U**,**V**); *ttn.2* and *ttn.1* N2A are also expressed (**W**,**X**), with stronger concentration near somite borders (red arrows). **Y** and **Z**, Wholemount *in situ* hybridization in 24 hpf embryos using riboprobe for *cronos* shows a severe reduction of Cronos expression in genotyped e129-/- mutants (**Y**) but not in e232-/- mutants (Z) when compared with siblings. **AA.** Wholemount *in situ* hybridization in 22 ss (somite stage) wild type embryos using riboprobe for *myom2a,* lateral view (top panel) and dorsal view (bottom panel), anterior to left. Expression is typical of differentiated fast skeletal muscle and lacking in the tail (arrow) as well as in the developing heart (arrowheads). **AB.** Wholemount *in situ* hybridization at 48 hpf in genotyped e232-/- mutants and sibling embryos for *ttn.1* kinase riboprobe, lateral view, anterior to left and high magnification of the heart in ventrolateral view, showing similar mRNA levels in somites and heart. All scale bars=100µm.

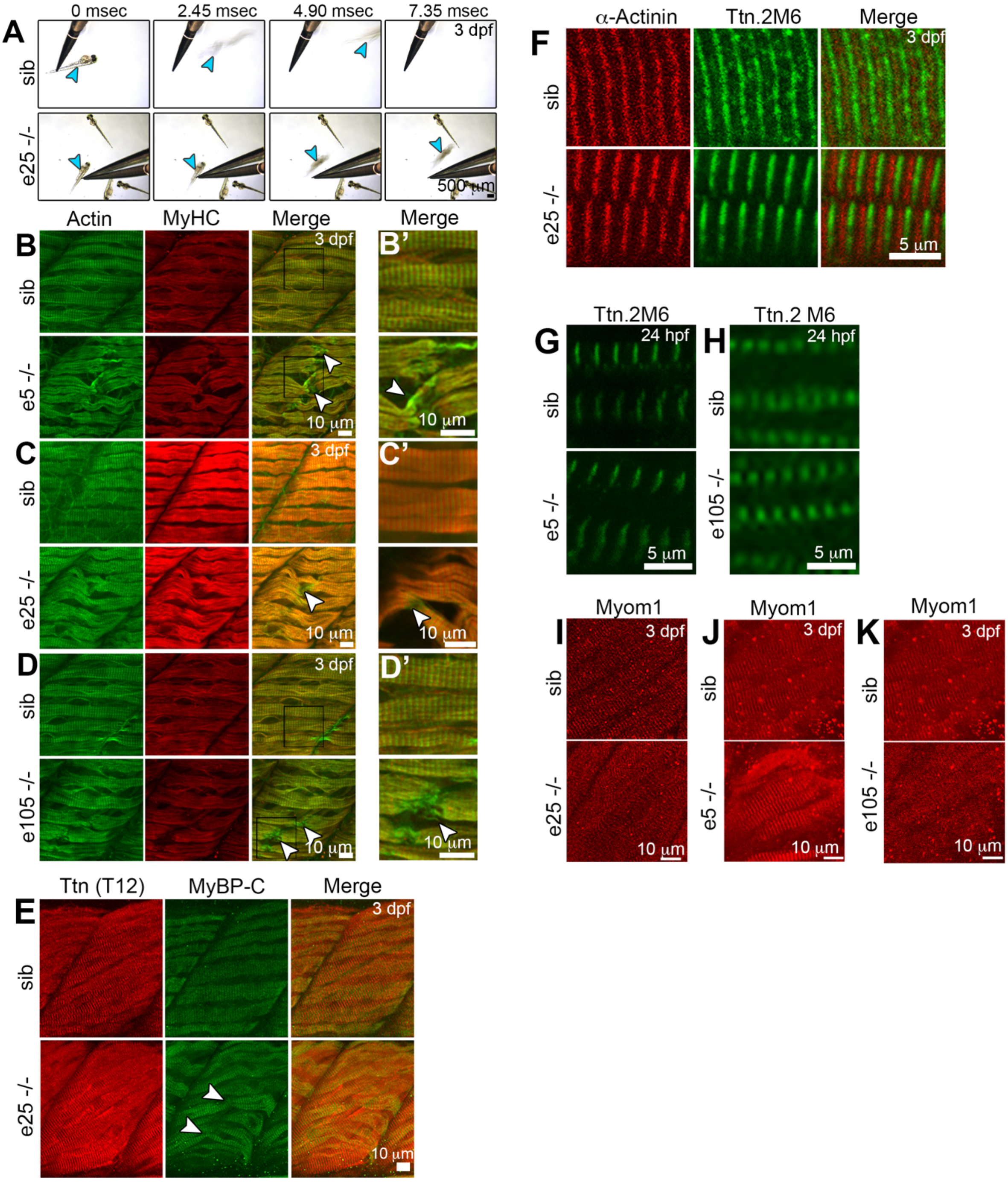

A
0 msec
2.45 msec
4.90 msec
7.35 msec
3 dpf
sib
e25 -/-
500 µm
Actin
MyHC
Merge
Merge
B
B'
C
C'
D
D'
e5 -/-
e105 -/-
10 µm
E
Ttn (T12)
MyBP-C
F
α-Actinin
Ttn.2M6
5 µm
G
H
Ttn.2 M6
24 hpf
I
J
K
Myom1

Figure S7. Homozygous embryos with *ttn.2* truncation sites proximal to the *cronos* promoter have abnormalities in muscle fiber structure and motility. A, Sequential brightfield images (2.45 msec apart) of 3 dpf e25-/- and sibling embryos showing response to mechano-stimulation. Each fish (blue arrowhead) is touched with forceps at 0 msec and tracked. In response to touch, e25-/- embryos respond but move more slowly and only a short distance compared with siblings. **B** through **D**, Confocal stacks of somitic slow muscle of 3 dpf e5-/- (**B**), e25-/- (**C**) and e105-/- (**D**) embryos and siblings immunostained for actin and myosin heavy chain (MyHC), ventral view, anterior to top. Mutants show variable fiber breakage and damage (white arrowhead) with greater effects in MyHC than actin. Higher magnification of the black box region (**B',D'**) or different fibers (**C'**) is shown. **E** and **F,** Immunofluorescence staining for Ttn T12 and myosin binding protein C (MyBP-C), α-actinin. and Ttn.2 M6 in 3dpf e25-/- and sibling control embryos, shown in lateral view, anterior to left. Slow sarcomeres in e25-/- embryos show strong MyBP-C and Ttn T12 staining but breaking points in fibers are evident (**E**, white arrowheads). High magnification shows α-actinin and Ttn.2 M6 (likely from Cronos) in e25-/- fibers (**F**). **G** and **H,** Immunofluorescence staining for Ttn.2 M6 in slow muscle fibres of 1 dpf e5-/- (**G**) and e105-/- (**H**) embryos and siblings. Staining patterns are normal in the mutant embryos, presumably detecting Cronos. **I** through **K**, Immunofluorescence staining for myomesin-1 (Myom1) in fast muscle fibers of 3 dpf e25-/- (**G**), e5-/- (**H**) and e105-/- (**I**) embryos and siblings shows normal striation patterns.

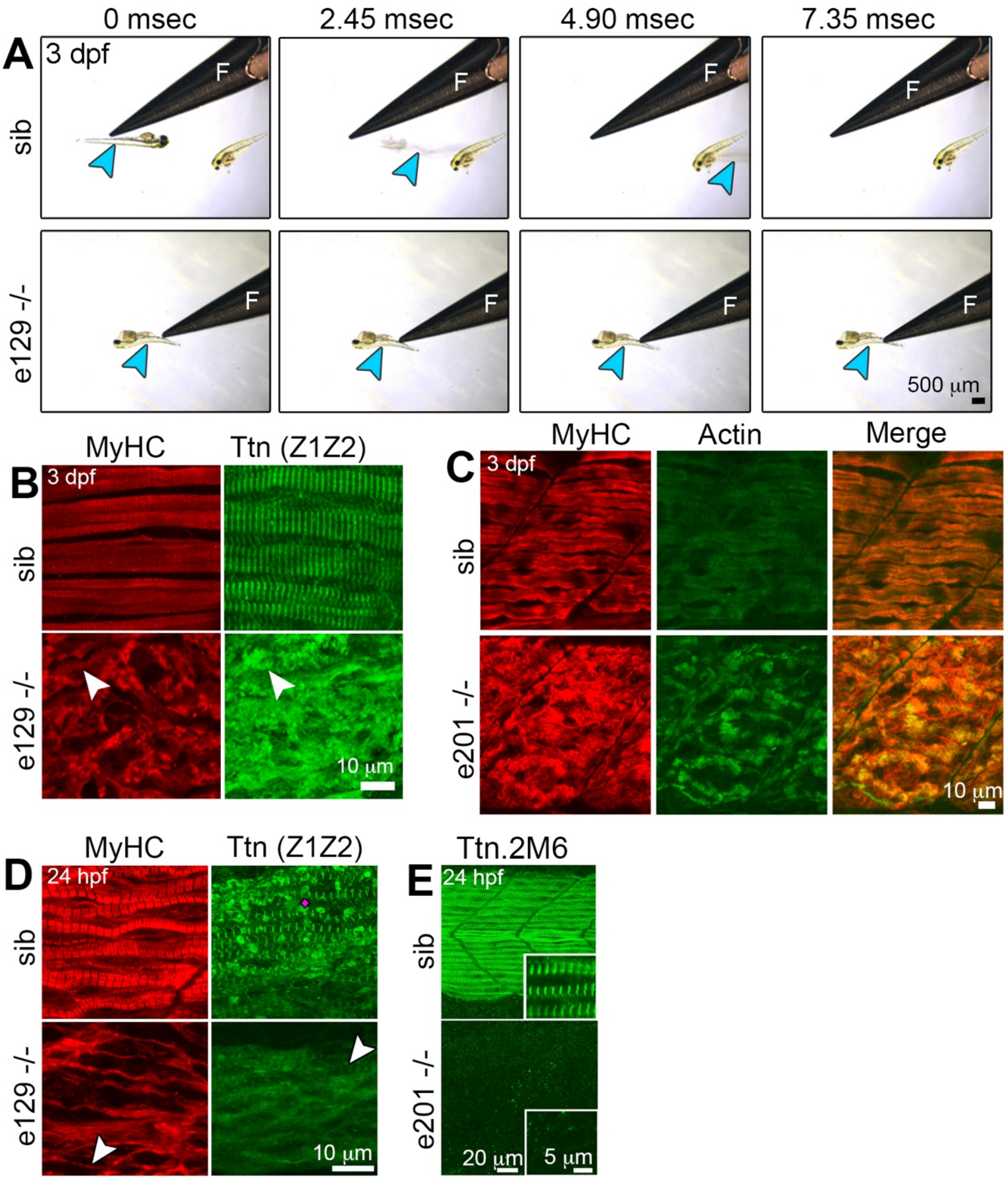

Figure S8. Homozygous embryos with *ttn.2* truncation sites distal to the *cronos* promoter have severe abnormalities in muscle structure and function. A, Sequential brightfield images (2.45 msec apart) of 3 dpf e129-/- and sibling (sib) control embryos after mechano-stimulation. Each fish (blue arrowhead) is touched with forceps at 0 msec and tracked. e129-/- mutants are paralyzed and do not respond to touch. **B** through E, Confocal stacks of somitic muscle of e129-/- (**B,D**) and e201-/- (**C,E**) and sibling control embryos at 24 hpf (**D,E**) or 3 dpf (**B,C**) immunostained for titin Z1Z2, myosin heavy chain *(*MyHC), actin, and Ttn.2 M6, ventral view, anterior to top. MyHC, actin and titin are all disorganized and aggregated in e129-/- and e201-/- embryos (**B,C**). At 24 hpf, fibers in e129-/- embryos are immature and show lack of organized sarcomeres (**D**). No Ttn.2 M6 is detected in e201-/- mutants (**E**).

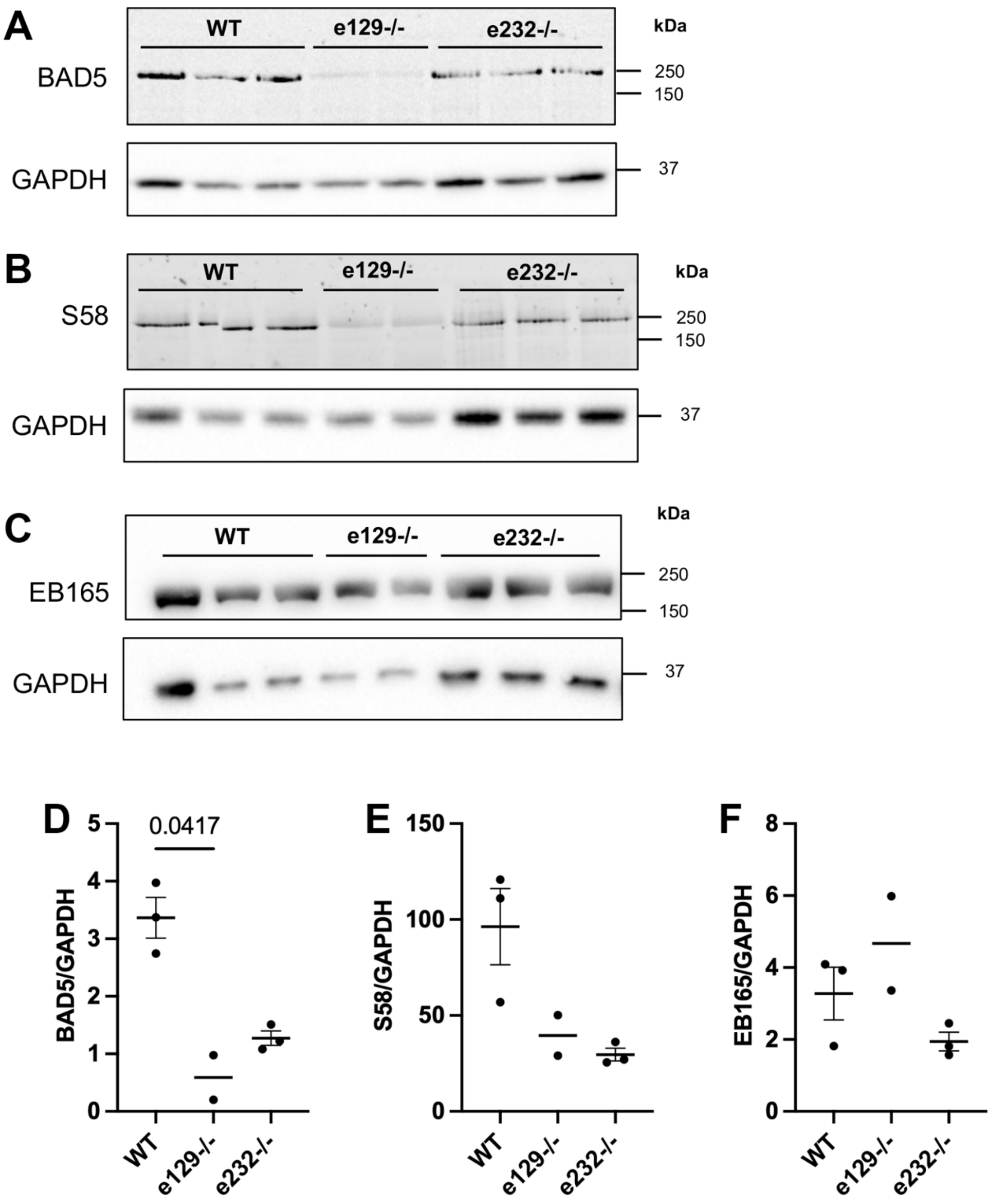

Figure S9. Evaluation of MyHC levels by western blot. A-C, Western blot analysis of MyHC levels from wild type (n=3), e129-/- (n=2) and e232-/- (n=3) tails of 5 dpf embryos using BAD5 (A, slow MyHC), S58 (B, slow MyHC) and EB165 (fast MyHC) and GAPDH (control) antibodies. **D-F,** Quantifications of MyHC western blot bands by normalising to GAPDH. Statistical comparisons were performed using Kruskal-Wallis test with Dunn's multiple comparison test, mean ± SEM.

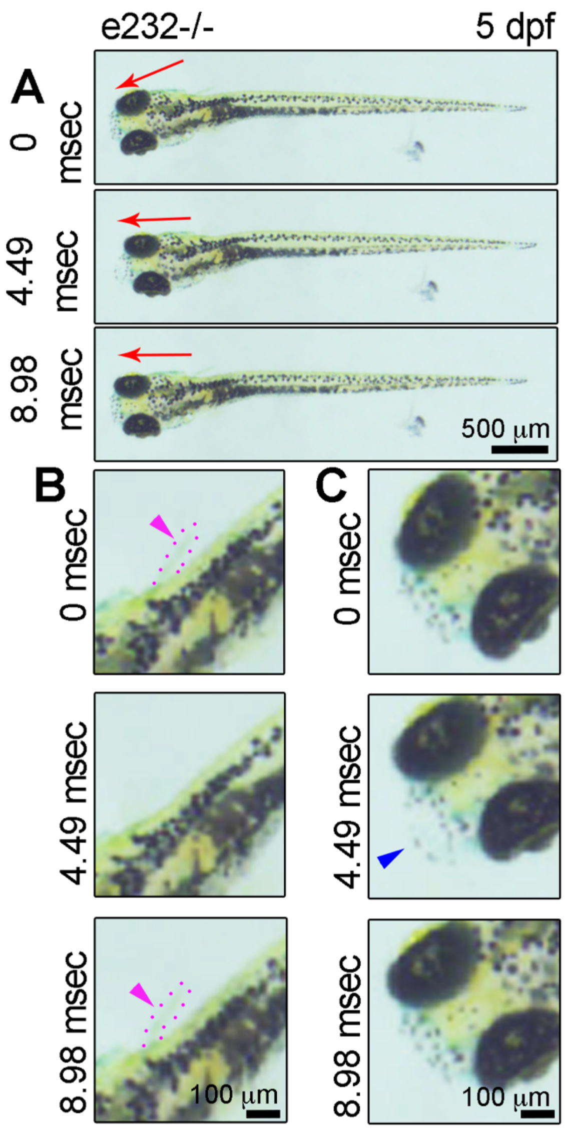

Figure S10. *ttn.2* e232-/- embryos maintain motility of various muscles. Sequential brightfield images (4.49 msec apart) of *ttn.2* e232-/- embryos show eye (**A**), jaw (**B**), and fin (**C**) movement at 5 dpf. Red arrows indicate change in eye direction (**A**). Magenta arrowheads (**B**) indicate pectoral fin movement (outlined by magenta dotted line). Opening of jaw is indicated with blue arrowhead (**C**).

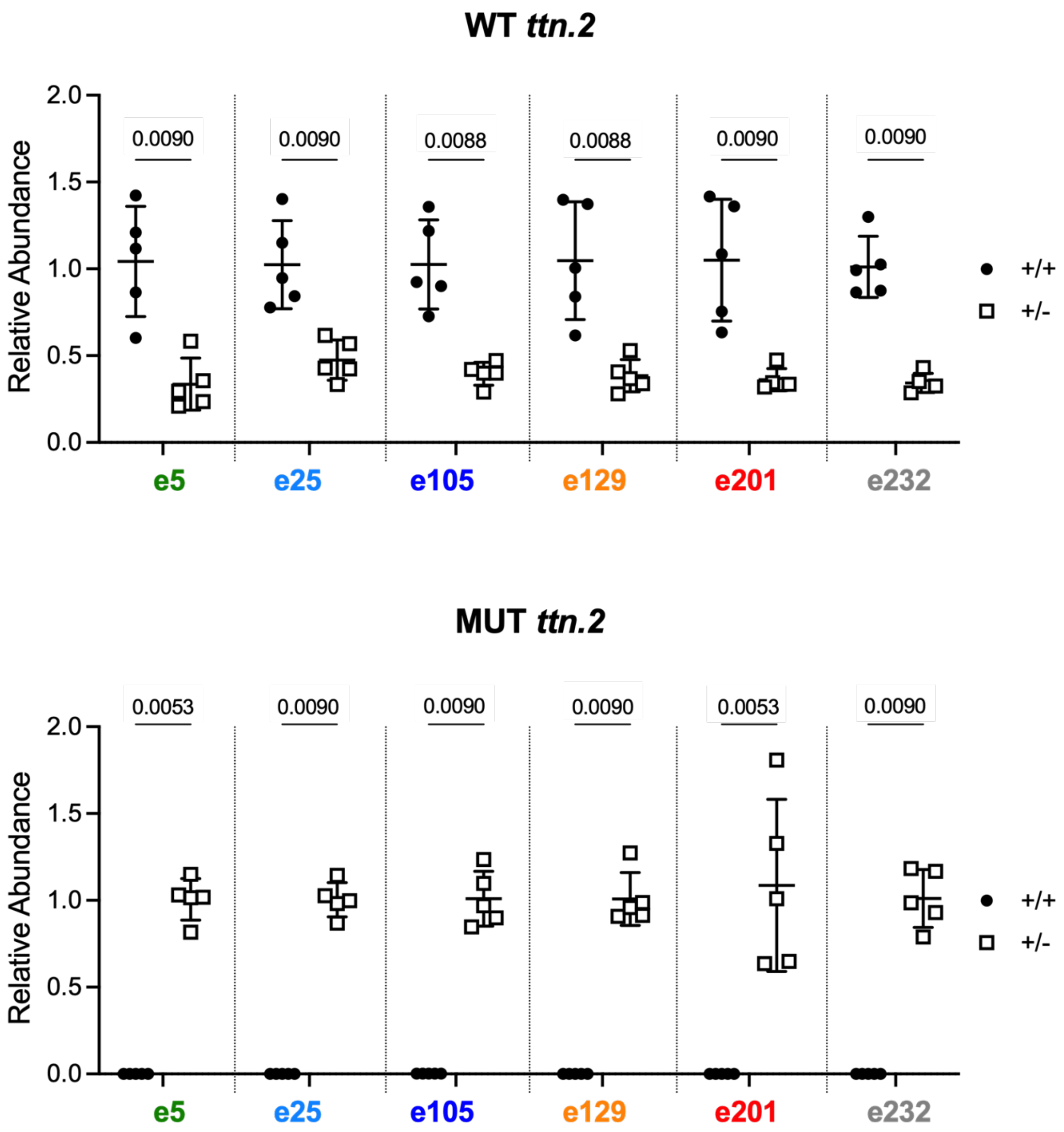

Figure S11. *ttn.2* transcript expression in adult heart. qPCR analysis of heart tissue from adult wild-type (WT; +/+) and heterozygous (MUT; +/-) *ttn.2* fish using WT-specific primers. Data were normalized to house-keeping genes *tmem50a* and *ube2a* and expressed as absolute levels of abundance.

Scheirer Ray Hare test with Dunn's multiple comparisons (genotype effects only, 6 comparisons made), adjusted p-values (Bonferroni's correction), mean ± SD, n=5 per genotype per line.

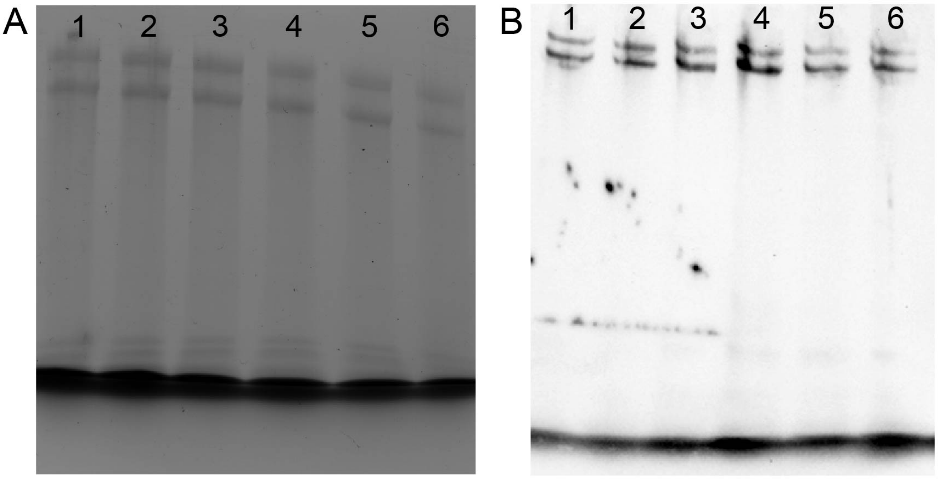

Figure S12. **Evaluation of titin levels in adult zebrafish heart.** Cardiac titin protein levels were evaluated using Coomassie staining following SDS-PAGE (**A**) or staining of PVDF membranes using an antibody targeting the Ttn N-terminus (**B**). Band densitometry showed no significant difference in titin protein levels across all 5 lines. There was no clear evidence of truncated titin protein on either Coomassie stained gels or Ttn antibody-stained PVDF membranes. Lanes: (1) wild-type, (2) e5+/-, (3) e25+/-, (4) e105+/-, (5) e129 +/-, (6) e201+/.

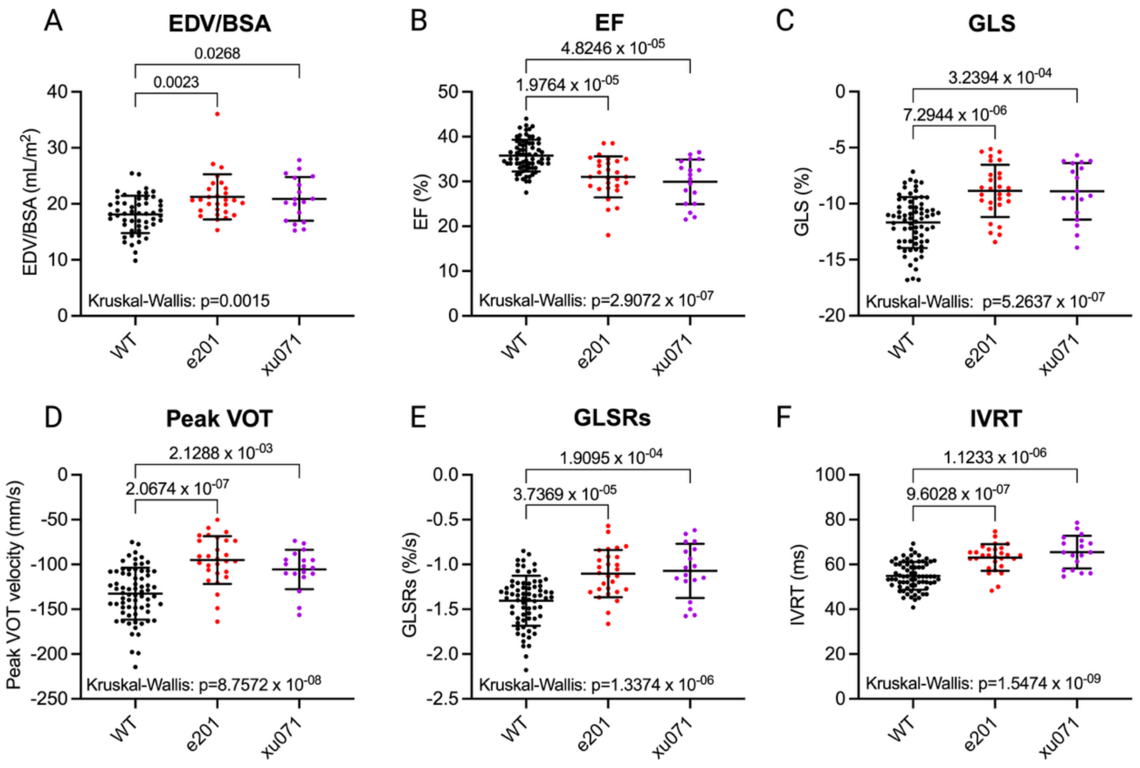

Figure S13. Comparison of ventricular size and function in adult heterozygous *ttn.2* and *ttn.2/ttn.1* double mutant zebrafish. Cardiac function was assessed in 9-month-old wild-type (WT), heterozygous single mutant (e201+/-), and heterozygous double mutant (xu071) fish using high frequency echocardiography. **A,** shows indexed ventricular end-diastolic volume (EDV/BSA); **B**, ejection fraction (EF); **C**, global longitudinal strain (GLS); **D**, peak ventricular outflow tract velocity (VOT); **E**, global longitudinal strain rate during systole (GLSRs); **F**, isovolumic relaxation time (IVRT). Kruskal-Wallis test and Dunn's multiple comparisons testing against WT group (2 comparisons), adjusted p-values (Bonferroni correction), mean ± SD, WT (n=70), e201 (n=28), xu071 (n=18).

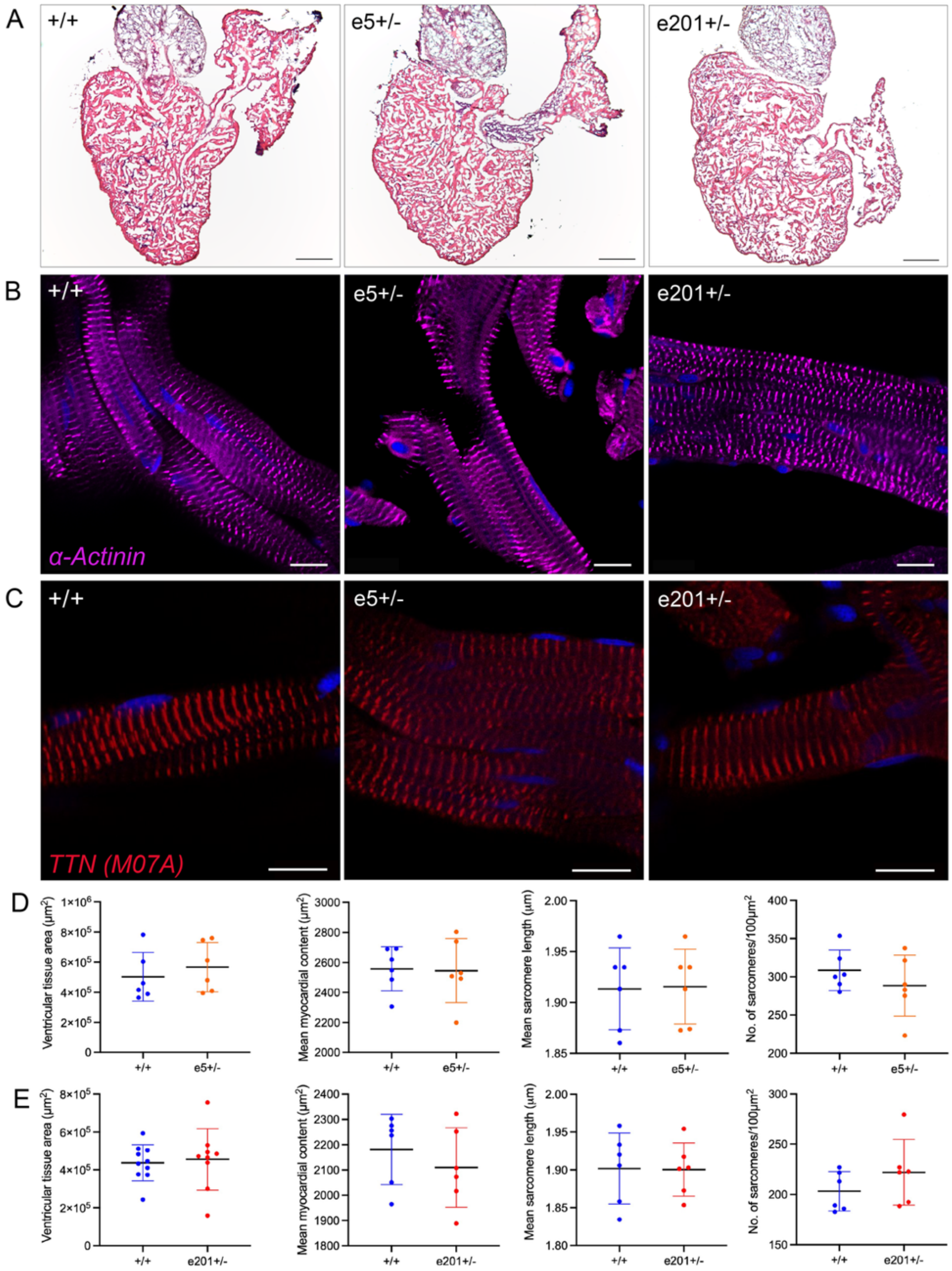

Figure S14. Normal cardiac morphology and sarcomeric structure in adult heterozygous *ttn.2* zebrafish. Semi-quantitative analysis of adult heart tissue stained with hematoxylin and eosin (**A**) showed no significant differences in the amount of ventricular trabeculation, assessed by ventricular tissue area or mean myocardial content, in e5+/- (**D**) or e201+/- fish (**E**) relative to wild-type (+/+) siblings (n=6 hearts per genotype for all comparisons except for e201 ventricular area, where WT n=10, e201+/- n=9). In ventricular tissue sections stained with α-actinin (sarcomere Z-disk marker) (**B**), or an N-terminal Ttn antibody (**C**), there was no evidence of reduced sarcomere number or abnormal periodicity (**D, E**) in the hearts of e5+/- (**D**) or e201+/- (**E**) relative to wild-type siblings. Scale bars: 100 µm. Data were obtained using image segmentation, with values averaged from 5 sections (ventricular tissue area) or 5 regions of interest (mean myocardial content, mean sarcomere length, number of sarcomeres) per heart, n=6 hearts. Unpaired Mann-Whitney U tests, mean ± SD.

Supplementary Table 2. Primers used for genotyping of mutant zebrafish lines.

**Allele**	**PCR primer 1**	**PCR primer 2**	**Restriction Enzyme**
ttn.2 e5	TTGTTCAAGATGGAGACCTTTACA	GAAAACTTCAGACACGTTACCTG	MboII
ttn.2 e25 (kg148)	TTGTTGAATGGCTCCATGATGGC	CAATACTCTGACATCTCCAGAATC	BfaI
ttn.2 e105	TGCCTACAGAAATTCTTTATGGTTGT	AGAGAAAACAAAAACAATCTTCTTCAA	TaaI
ttn.2 e129 (kg149)	GAGGCTGAGTGGTTCTACAATGACA	ATCTTGCTGCCGCCATCACT	Eam1104I
ttn.2 e201 (vcc14)	AACCTATGAGGTTGCTTCAG	ACGTAGGTCTGTGACTCTGC	HindIII
ttn.2 e232 (kg150)	CCTGGCAATGATGGTGGAAGTG	GTCCATTACGTGCAAGTTCCTCAGA	NmuCI
ttn.1 e7 (kg184)	GCAGCTCCACGCCATCAGTC	CAAATGACCTTCCCACTGCTGTTC	BpmI

Supplementary Table 2. Primers used for qPCR analysis.

	**Primer name**	**Sequence**
ttn.2 z-disk	*ttna*_e6_F	TGCCACAATGCAAATGCAGG
	*ttna*_e7_R	AGGGGACTTGACAGGTCTGA
ttn.2 I-band	*ttna*_e103_F	GCAAAGCTTGGCAACAAGGA
	*ttna*_e104_R	TCCTGGATCGTTGCTGATCG
ttn.2 A-band	*ttna*_e154_F	ACAAACTGGCAGAAATGCTC
	*ttna*_e154_R	GTCGACTTTCTCGAGGTTCA
ttn.2 M-band	*ttna*_e231-232_F	CAATCTCGACAAGTATGAACC
	*ttna*_e231-232_R	CAGTGATGGTACGTGTTTGAC
Cronos	*ttna*_Cronos-F	GGAAAGCGCTATGTGGATTT
	*ttna*_Cronos-R	CCTAAGGGTTAGCGTGTGAGA
ttn.1 A-band	*ttnb*_e115_F	TGGGAAATGCCTCTTATTGA
	*ttnb*_e115_R	GATGACTTCGCCCAGAACTA
Housekeeping gene	*tmem50a*_F	ATTTGTGTTGATTTTGCTGTCG
	*tmem50a*_R	TTCTGTTGCAAACTCTGAGGAA
Housekeeping gene	*ube2a*_F	TGACTGTTGACCCACCTTACAG
	*ube2a*_R	CAAATAAAAGCAAGTAACCCCG
Allele-specific primers:		
WT allele specific primers:	*ttna*_e5_WT-F	GCTTGTTCAGGGTGAGGAAG
	*ttna*_e6_WT-R	GGTGTCGGGCCCTTAACAT
	*ttna*_e25_WT-F	AATGGTTGTTGAATGGCTCC

	*ttna*_e25_WT-R	GTAATTGACTGTCCTCTACC
	*ttna*_e105_WT-F	GAGATCACACTTACAGTCAAG
	*ttna*_e106_WT-R	CCGAGAGTGAGTTTTGCTGT
	*ttna*_e129_WT-F	AGGACCCTAAGAAATCAGAAG
	*ttna*_e129_WT-R	CTCTGCAAGCCAGTTACAGT
	*ttna*_e201_WT-F	GTTCAAAGTTACAAAGCTTCTAA
	*ttna*_e201-WT-R	ATTTCTAGATCTTGAGGTGGG
	*ttna*_e232-WT-F	ATCGCTTTGGCATGGATA
	*ttna*_e232-WT-R	CTTTGGTAGTCACAGGGT
MUT allele specific primers:	*ttna*_e4_MUT-F	ACAGAGAGGGAGCCGAGATT
	*ttna*_e5_MUT-R	CTTTTTTGCAGGTACGGCTTA
	*ttna*_e25_MUT-F	GGTTTGGACCTGTGCACTTT
	*ttna*_e25_MUT-R	TGGTAATTGACTGTCCTCTAGT
	*ttna*_e104_MUT-F	AGACATCAAAACACAAGCC
	*ttna*_e105_MUT-R	GGTTGAGCATTCTAAGTGTG
	*ttna*_e129_MUT-F	TGGTGACAGATGCTGGAAAG
	*ttna*_e129_MUT-R	GATGATCTTATAGCGTCCCTGA
	*ttna*_e201_MUT-F	CCATTGACTGTTTCTGGTGT
	*ttna*_e201-MUT-R	ATTCATTTCCTTTTTGTAACT
	*ttna*_e232-MUT-F	CTGCCAAAGAAGACTACCAA
	*ttna*_e232-MUT-R	GCCAAACTGTCCATTACGTG

Supplementary Table 3. Primers used for in situ hybridization (ISH) probes.

**ISH probe**	**Primer name**	**Sequence**
ttn.2 kinase	*ttna*_kinase_FWD	CCTGGCAATGATGGTGGAAGTG
ttn.2 kinase	T3-*ttna*_kinase_REV	GGATCCATTAACCCTCACTAAAGGGAATGCATTGTCCTCCCTGTCTTCACT
ttn.1 kinase	*ttnb*_kinase_FWD	CTTGGCCGTGGACAGTTTGGTAT
ttn.1 kinase	T3-*ttnb*_kinase_REV	GGATCCATTAACCCTCACTAAAGGGAAGCAGGAGAAGGACGAGGGACTACT
ttn.2 N2A	*ttna*_N2A_FWD	ACACCATCCAAGCAGAAAAGC
ttn.2 N2A	T3-*ttna*_N2A_REV	GGATCCATTAACCCTCACTAAAGGGAATTCCTTCTTCAAAAACTCG
ttn.1 N2A	*ttnb*_N2A_ FWD	ATCAAAGCAGAAAAGTCCTAAG
ttn.1 N2A	T3-*ttnb*_N2A_REV	GGATCCATTAACCCTCACTAAAGGGAATCACCCTTCTCCTTCTTCAG
ttn.2 N2B	*ttna*_N2B_ FWD	AAGCATACAGTGTCAGTGGATTTACAGC
ttn.2 N2B	T3-*ttna*_N2B_REV	GGATCCATTAACCCTCACTAAAGGGAAGTCTGAGGATACTCGCCTTC
ttn.1 N2B	*ttnb*_N2B_ FWD	GGAGGTTATAGTTAGTGGTCC
ttn.1 N2B	T3-*ttnb*_N2B_REV	GGATCCATTAACCCTCACTAAAGGGAAGAGCAAAACAGGCTGCGAGACA
ttn.2 cronos	*Cronos*_FWD	FGAAAGCGCTATGTGGATTTGTAG
ttn.2 cronos	T7-cronos_REV	GCGTAATACGACTCACTATAGCTTTGGATTCAGGAGAAGACATTG
myom2a	*myom2a*-FWD	CAGTGCACACCAGAGGGAATCAGA
myom2a	T7-*myom2a*-REV	TAATACGACTCACTATAGGGAGAGAGGCCTTTGCGATGATTTTTGAC

Supplementary Table 4. Antigen sequences for antibody production.

Antibody	Antigen amino acid sequence
Ttn.2 M6	SPVPSVKSPEPLVKSPVPSLKSPEPSVKSPVPSVKSPEPQIKSPEPTGIKSPEPRIKSPEG<br>IKSPFRVKSPEPATSLQRVKSPPPLKSPEPTTPQGVKSPIASPPRVKSPPPIKSPEPIASP<br>LRVKSPTGLKSPEPQRAKSPPTVKSPEPIMSPKRMKSPLTVKSPTPSKEAPPKIIQQLKAE<br>AFEDKIRMIFVAESSLREVVWYKDSRKLSQSSHYQIHSSADGTCCLYISDVSEDDQGEYSC<br>EIISEGGAVSRTSFSFVGQVFQAIYTKVTAFVAAHKAVQESVSSKIQGGSEMVI
Ttn.1 M8-M9	AALEGKSELTEEIVKKENTYEEVQSYTEIKASKTQMTISQGQTVTLRASIPEASDVKWILN<br>GAELSNSESYRYGVSGSDHTLTIKSISHHDQGILTCEARTEQGVVKCQFDMTVSATHSGSP<br>SFLVQPHSQNVNEGQNVTFTCEITGEPSPEVEWLKDNAVISITSNMKLSRSKNVYTLEIHN<br>ATIEDSGKFTVKAKNKFGQCSATASLNVLTLVEEPARMIIMEKASDATSMQGSFSAKHVVS<br>KMQESSFSSSSM

Supplementary Table 5. Zebrafish *ttn.2* exons targeted in the mutant lines evaluated and corresponding human *TTN* exons.

ttn.2 allele		Zebrafish			Human			
	Variant location	Exon number*	Exon size (bp)	Homology with human exon (%)	Exon number†	Exon size (bp)	Exon PSI‡ (GTeX, %)	Exon PSI (DCM, %)
e5	Z-disk	5	89	68.6	5	86	100	100
e25	Proximal I-band	25	1697	71.3	28	1694	100	100
e105	Distal I-band	105	276	66.7	227	276	98	100
e129	Proximal A-band	129	270	64.1	251	270	89	100
e201	Mid A-band	201	17103	69.3	326	17106	95	100
e232	Distal A-band	232	5717	62.2	358	5609	100	100

* Zebrafish exon numbering as described in Seeley et al [11] (accession no. DQ649453).
† Human exon numbering in accordance with inferred complete human *TTN* meta-transcript (NM_001267550.2).
‡ Exon percent splice-in (PSI) scores in adult heart tissue in the Genotype-Tissue Expression (GTeX) database and in patients with dilated cardiomyopathy (DCM) [3].

Supplementary Table 6. Echocardiographic parameters in adult (12 month-old) *ttn.2* zebrafish.

**Parameter**	+/+ (n=68)	**e5+/-** (n=30)	*p-value*	**e25+/-** (n=15)	*p-value*	**e105+/-** (n=17)	*p-value*	**e129+/-** (n=15)	*p-value*	**e201+/-** (n=15)	*p-value*	*ANOVA p-value (approximate)*
Body weight (g)	0.67 ± 0.08	0.65 ± 0.08	1.00	0.66 ± 0.05	1.00	0.69 ± 0.08	0.97	0.66 ± 0.09	1.00	0.67 ± 0.10	1.00	0.646
Heart rate (bpm)	136 ± 19	133 ± 17	1.00	144 ± 17	0.86	136 ± 15	1.00	122 ± 17	0.16	136 ± 14	1.00	0.022
EDV/BSA (mL/m^2)	18.7 ± 3.3	18.5 ± 4.6	1.00	20.8 ± 4.5	0.48	18.6 ± 3.1	0.050	21.3 ± 5.5	0.64	22.6 ± 4.1	0.0087	0.056
ESV/BSA (mL/m^2)	11.7 ± 2.4	11.4 ± 2.2	1.00	12.8 ± 2.6	1.00	13.2 ± 2.6	0.23	13.6 ± 3.4	0.37	16.5 ± 3.9	2.659×10^{-5}	2.014×10^{-5}
EF (%)	36.0 ±3.8	35.4 ± 4.4	1.00	31.7 ± 5.2	0.024	32.6 ± 4.9	0.050	31.3 ± 4.8	0.005	29.1 ± 6.2	0.0002	8.182×10^{-6}
GLS (%)	-12.8 ± 2.7	-13.1 ± 2.5	1.00	-10.8 ± 2.5	0.09	-12.0 ± 2.6	1.00	-10.2 ± 2.5	0.014	-9.2 ± 3.0	0.001	4.019×10^{-5}
GLSRs (%/s)	-1.37 ± 0.27	-1.45 ± 0.38	1.00	-1.48 ± 0.37	1.00	-1.31 ± 0.34	1.00	-0.99 ±0.19	5.997×10^{-5}	-1.10 ± 0.19	0.006	2.641×10^{-6}
Peak VOT (mm/s)	-121.3 ± 24.7	-112.1 ± 22.0	1.00	-131.8 ± 35.8	1.00	-116.7 ± 24.1	1.00	-89.7 ± 29.5	0.0005	-97.6 ± 24.1	0.020	2.538×10^{-4}
VOT-VTI (mm)	12.95 ± 4.40	13.06 ± 3.07	1.00	11.04 ± 3.13	0.55	13.27 ± 4.21	1.00	9.36 ± 3.64	0.006	11.07 ± 2.43	1.00	0.0043
GLSRa (%/s)	1.42 ± 0.50	1.45 ± 0.54	1.00	1.09 ± 0.33	0.046	1.25 ± 0.38	0.85	0.99 ± 0.34	0.005	1.10 ± 0.31	0.119	0.0013
Atrial area/ BSA(cm^2/m^2)	6.7 ± 1.5	6.8 ± 1.9	1.00	6.2 ± 1.7	0.83	7.2 ± 1.0	1.00	8.0 ± 1.7	0.076	7.9 ± 1.0	0.023	0.0037
E (mm/s)	44 ± 15	38 ± 11	0.40	48 ± 13	1.00	38 ±16	0.63	37 ± 15	0.36	26 ± 8	1.521×10^{-5}	2.192×10^{-5}
A (mm/s)	294 ± 57	280 ± 47	1.00	287 ± 47	1.00	266 ± 49	0.42	282 ± 50	1.00	232 ± 32	9.094×10^{-5}	0.0013
E/A	0.15 ± 0.06	0.13 ± 0.04	0.58	0.17 ± 0.04	0.949	0.14 ± 0.05	1.00	0.13 ± 0.05	0.87	0.11 ± 0.04	0.022	0.0141
IVRT	54 ± 8	58 ± 11	0.27	56 ± 6	1.00	68 ± 8	1.397×10^{-6}	65 ± 10	0.007	70 ± 12	1.306×10^{-5}	1.268×10^{-8}

Groups compared using Kruskal-Wallis' non-parametric one-way ANOVA with Dunn’s multiple comparisons tests (5 comparisons). P-values were corrected for multiple comparisons using Bonferroni’s correction. All other P-values refer to differences between each *ttn.2* line to wild-types (+/+). Data presented as mean ± SD. A, denotes peak velocity of blood inflow across atrioventricular valve during late diastole; bpm, beats per minute; E, peak velocity of blood inflow across atrioventricular valve during early diastole; EDV/BSA, ventricular end-diastolic volume indexed to body surface area; EF, ejection fraction; ESV, ventricular end-systolic volume; GLS, global longitudinal strain; GLSRa, global longitudinal strain rate during atrial contraction; GLSRs, peak systolic global longitudinal strain; IVRT, isovolumic relaxation time; VOT, peak velocity of blood outflow from across bulbo-ventricular valve; VOT-VTI, ventricular outflow tract-velocity time integral.

Major Resources Table

Animals (in vivo studies)

Species	Vendor or Source	Background Strain	Sex	Persistent ID / URL
Zebrafish	ZIRC	wild type TL (Tüpfel long fin)	Unselected embryos; male adults.	RRID:ZIRC_ZL86
Zebrafish	ZIRC	wild type AB	Unselected embryos; male adults.	RRID:ZIRC_ZL1
Zebrafish	This work	*ttn.1*^{kg184}	Unselected embryos; male adults.	kg184
Zebrafish	This work	*ttn.2*-e25 *ttn.2*^{kg148}	Unselected embryos; male adults.	kg148
Zebrafish	This work	*ttn.2*-e129 *ttn.2*^{kg149}	Unselected embryos; male adults.	kg149
Zebrafish	This work	*ttn.2*-e232 *ttn.2*^{kg150}	Unselected embryos; male adults.	kg150
Zebrafish	This work	*ttn.2*-e5	Unselected embryos; male adults.	
Zebrafish	This work	*ttn.2*-e105	Unselected embryos; male adults.	
Zebrafish	This work and ref.	*ttn.2*-e201 *ttn.2*^{vcc14}	Unselected embryos; male adults.	https://www.ahajournals.org/doi/full/10.1161/CIRCGEN.118.002135 vcc14
Zebrafish	Xiaolei Xu	*ttn*^{xu071}	Unselected embryos; male adults.	ZDB-FISH-170308-16, https://doi.org/10.1242/dev.139246

Genetically Modified Animals

	Species	Vendor or Source	Background Strain	Other Information	Persistent ID / URL
**Parent - Male**					
**Parent - Female**					

Antibodies

Target antigen	Vendor or Source	Catalog #	Working conc.	Lot # (preferred but not required)	Persistent ID / URL
actin (Actc1a), rabbit polyclonal	GeneTex	#GTX124462	1:100 (IF)		RRID:AB_11164112
α-actinin (clone EA-53), mouse monoclonal IgG1	SIGMA	# A7811	1:500 (IF)		RRID:AB_476766

DOI [to be added]

Myom1 (Myomesin) B4, mouse monoclonal IgG1	J.C. Perriard		1:100 (IF)		RRID:AB_760349
Myom2 AA259, mouse monoclonal IgGA	Dieter Fürst		1:5 (IF)		https://doi.org/10.1242/jcs.106.1.319
A4.1025 (pan myosin), mouse monoclonal IgG2a	Simon Hughes		1:10 (IF)		RRID:AB_528356
mouse anti-myosin heavy chain I (BAD5)	Developmental Studies Hybridoma Bank (DHSB)		1:20 (WB)		Schiaffino et al., 1989<br>RRID: AB_2235587
mouse anti-slow myosin heavy chain 1 (S58)	Developmental Studies Hybridoma Bank (DHSB)		1:20 (WB)		Miller et al., 1985<br>RRID:AB_528377
mouse anti-fast myosin heavy chain (EB165)	Simon Hughes		1:50 (WB)		Gardahaut et al., 1992<br>RRID:AB_531860
myosin binding protein C (MyBP-C) C0-C1, rabbit polyclonal	Mathias Gautel		1:200 (IF)		https://doi.org/10.1161/01.RES.82.1.124
fast MyLC, F310	DSHB		1:10 (IF)		RRID:AB_531863
titin (T12)	Dieter Fürst		1:50 (IF)		https://doi.org/10.1083/jcb.106.5.1563
titin (Z1Z2)	S. Labeit		1:100 (IF)<br>1:1000(WB)		https://doi.org/10.1083/jcb.143.4.1013
Ttn.2 M6			1:1000 (IF)<br>1:1000(WB)		This work
Ttn.1 M8-M9			1:2000 (IF)		This work
Titin (2F12)	Abnova	#H00007273-M07A	1:1000 (WB)		

DOI [to be added]

Secondary, Alexa Fluor™ 488 goat anti-mouse IgG(H+L)	Invitrogen	#A-11001	1:1000 (IF)		https://www.thermofisher.com/antibody/product/Goat-anti-Mouse-IgG-H-L-Cross-Adsorbed-Secondary-Antibody-Polyclonal/A-11001
Secondary, Alexa Fluor™ 488 goat anti-rabbit IgG(H+L)	Invitrogen	#A-11008	1:1000 (IF)		https://www.thermofisher.com/antibody/product/Goat-anti-Mouse-IgG-H-L-Cross-Adsorbed-Secondary-Antibody-Polyclonal/A-11008
Secondary, Alexa Fluor™ 488 goat anti-mouse IgG1	Invitrogen	#A-21121	1:1000 (IF)		https://www.thermofisher.com/antibody/product/Goat-anti-Mouse-IgG1-Cross-Adsorbed-Secondary-Antibody-Polyclonal/A-21121
Secondary, Alexa Fluor™ 555 goat anti-mouse IgG(H+L)	Invitrogen	#A-21422	1:1000 (IF)		https://www.thermofisher.com/antibody/product/Goat-anti-Mouse-IgG-H-L-Cross-Adsorbed-Secondary-Antibody-Polyclonal/A-21422
Secondary, Alexa Fluor™ 555 goat anti-mouse IgG1(g1)	Invitrogen	#A-21127	1:1000 (IF)		https://www.thermofisher.com/antibody/product/Goat-anti-Mouse-IgG1-Cross-Adsorbed-Secondary-Antibody-Polyclonal/A-21127
Secondary, Alexa Fluor™ 555 goat anti-rabbit IgG(H+L)	Invitrogen	#A-21428	1:1000 (IF)		https://www.thermofisher.com/antibody/product/Goat-anti-Rabbit-IgG-H-L-Cross-Adsorbed-Secondary-Antibody-Polyclonal/A-21428
Secondary, Alexa Fluor™ 568 goat anti-mouse IgG2a	Invitrogen	#A-21134	1:1000 (IF)		https://www.thermofisher.com/antibody/product/Goat-anti-Mouse-IgG2a-Cross-Adsorbed-Secondary-Antibody-Polyclonal/A-21134
Secondary, Alexa Fluor™ 633 goat anti-mouse IgG(H+L)	Invitrogen	#A-21050	1:1000 (IF)		https://www.thermofisher.com/antibody/product/Goat-anti-Mouse-IgG-H-L-Cross-Adsorbed-Secondary-Antibody-Polyclonal/A-21050
Secondary, Alexa Fluor™ 633 goat anti-rabbit IgG(H+L)	Invitrogen	#A-21071	1:1000 (IF)		https://www.thermofisher.com/antibody/product/Goat-anti-Rabbit-IgG-H-L-Highly-Cross-Adsorbed-Secondary-Antibody-Polyclonal/A-21071
Goat anti IgA FITC	Sigma-Aldrich	#F9384	1:25 (IF)		https://www.sigmaaldrich.com/GB/en/product/sigma/f9384?srsltid=AfmBOopGoENHR98kFc27j7t5dd6obftRkEsFuH_uWsZzF1QTpBMrMSNI
Cy3 AffiniPure Goat Anti Mouse IgG, Fcγ	Jackson ImmunoResearch Laboratories Inc.	#115-165-071	1:100 (IF)		RRID: AB_2338687

DOI [to be added]

fragment specific					
polyclonal goat anti-mouse immunoglobulins/HRP	Agilent	P0447	1:2000 (WB)		https://www.agilent.com/store/en_US/Prod-P044701-2/P044701-2
goat anti-rabbit IgG, H & L Chain Specific Peroxidase Conjugate	Merck Millipore	401315	1:2000 (WB)		https://www.sigmaaldrich.com/GB/en/product/mm/401315m
IRDye® 800CW donkey anti-mouse	LI-CORbio	926-32212	1:5000 (WB)		https://www.licorbio.com/support/contents/reagents/irdye-secondary-antibodies/800cw/donkey-anti-mouse-igg.html
Anti-mouse IgG-HRP	Cytiva	#NA9310	1:15000 (WB)		RRID:AB_772193
Sheep anti-digoxigenin Fab fragments Antibody, AP conjugated	Sigma (Roche)	#11093274910	1:5000 (ISH)		RRID:AB_514497

DNA/cDNA Clones

Clone Name	Sequence	Source / Repository	Persistent ID / URL

Cultured Cells

Name	Vendor or Source	Sex (F, M, or unknown)	Persistent ID / URL

Data & Code Availability

Description	Source / Repository	Persistent ID / URL
Proteomics data (wt, e129, e232)	Proteomics Identification Database (PRIDE)	Ref. 1-20250911-125240-2290747 (awaiting PXD number)

Other

Description	Source / Repository	Persistent ID / URL
RNeasy Micro Kit, ID 74004	Qiagen	https://www.qiagen.com/gb/products/discovery-and-translational-research/dna-rna-purification/rna-purification/total-rna/rneasy-kits?catno=74004
Superscript III First-Strand Synthesis System, #18080051	Invitrogen	https://www.thermofisher.com/order/catalog/product/18080051
EnGene Spy Cas9 NLS protein	New England Biolabs	https://www.neb.com/en-gb/products/m0646-engen-spy-cas9-nls

DOI [to be added]

#M0646T		
Alt-R CRISPR tracrRNA, #1072534	Integrated DNA Technologies	https://eu.idtdna.com/pages/products/crispr-genome-editing/alt-r-crispr-cas9-system
Alt-R CRISPR crRNA	Integrated DNA Technologies	https://eu.idtdna.com/pages/products/crispr-genome-editing/alt-r-crispr-cas9-system
Hoechst 33258	Sigma-Aldrich	https://www.sigmaaldrich.com/GB/en/product/sigma/94403
Epinephrine hydrochloride	Sigma-Aldrich	https://www.sigmaaldrich.com/GB/en/product/sigma/e4642
MboII	NEB	https://www.neb.com/en-gb/products/r0148-mboii
BfaI	NEB	https://www.neb.com/en-gb/products/r0568-bfai
TaaI	ThermoScientific	https://www.thermofisher.com/order/catalog/product/ER1362?SID=srch-srp-ER1362
Eam1104I	ThermoScientific	https://www.thermofisher.com/order/catalog/product/ER0232?SID=srch-srp-ER0232
HindIII	NEB	https://www.neb.com/en-gb/products/r0104-hindiii
NmuCI	ThermoScientific	https://www.thermofisher.com/order/catalog/product/ER1511
BpmI	NEB	https://www.neb.com/en-gb/products/r0565-bpmi
BL21-CodonPlus (DE3)-RIPL Competent Cells	Agilent	https://www.agilent.com/store/productDetail.jsp?catalogId=230280&catId=SubCat3ECS_228019

DOI [to be added]

ARRIVE GUIDELINES

The ARRIVE guidelines (https://arriveguidelines.org/) are a checklist of recommendations to improve the reporting of research involving animals. Key elements of the study design should be included below to better enable readers to scrutinize the research adequately, evaluate its methodological rigor, and reproduce the methods or findings.

Study Design

**Groups**	**Sex**	**Age**	**Number (prior to experiment)**	**Number (after termination)**	**Littermates (Yes/No)**	**Other description**
Group 1 (Control)						
Group 2						
Add more if needed						

Sample Size: Please explain how the sample size was decided Please provide details of any a *prior* sample size calculation, if done.

Inclusion Criteria

Exclusion Criteria

Randomization

Blinding

DOI [to be added]
