# Supplementary material for: Location-Dependent Differences in Cardiac and Skeletal Muscle Dysfunction Associated With Truncating Titin (ttn.2) Variants
Source: Circ Res. 2026 Jan 7;138(3):e325999. doi: 10.1161/CIRCRESAHA.124.325999 (PMC12854356; doi:10.1161/CIRCRESAHA.124.325999)

Unedited gels for representative cropped gels:

Full unedited gel for Figure S9A:

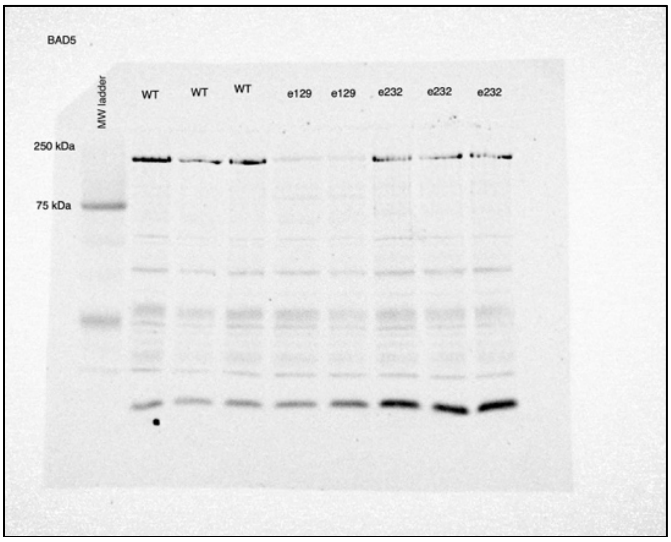

Full unedited gel for Figure S9B:

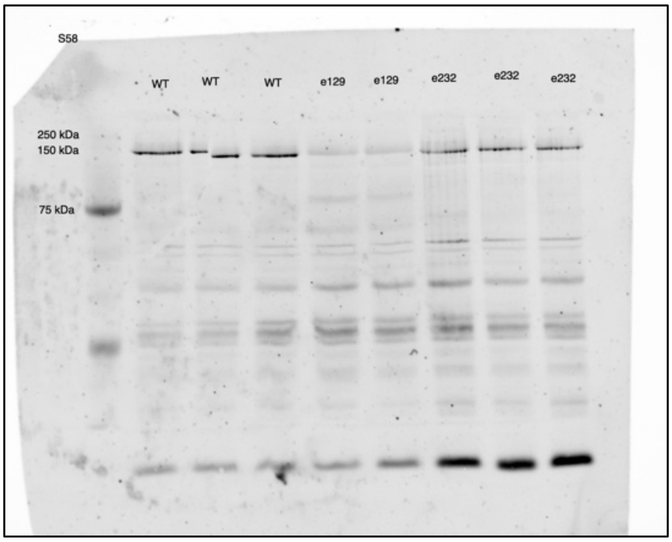

Full unedited blot for GAPDH Figure S9A,B:

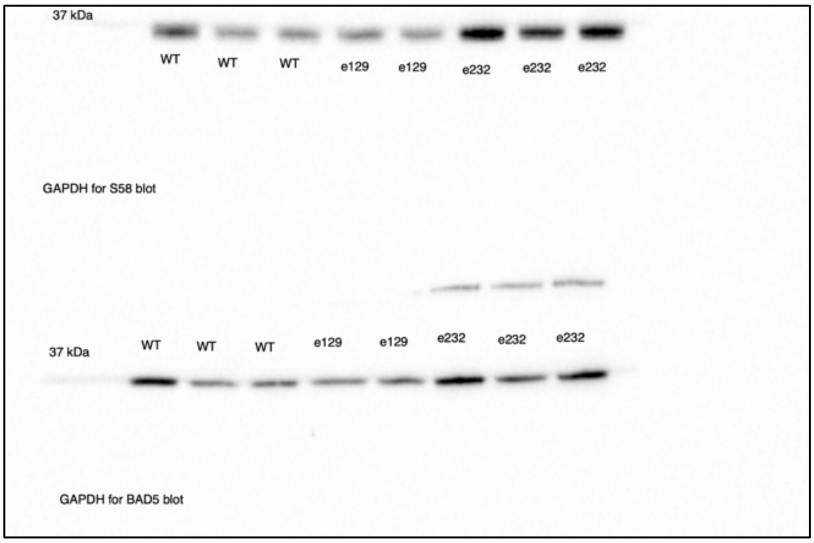

Full unedited gel for Figure S9C
Original:

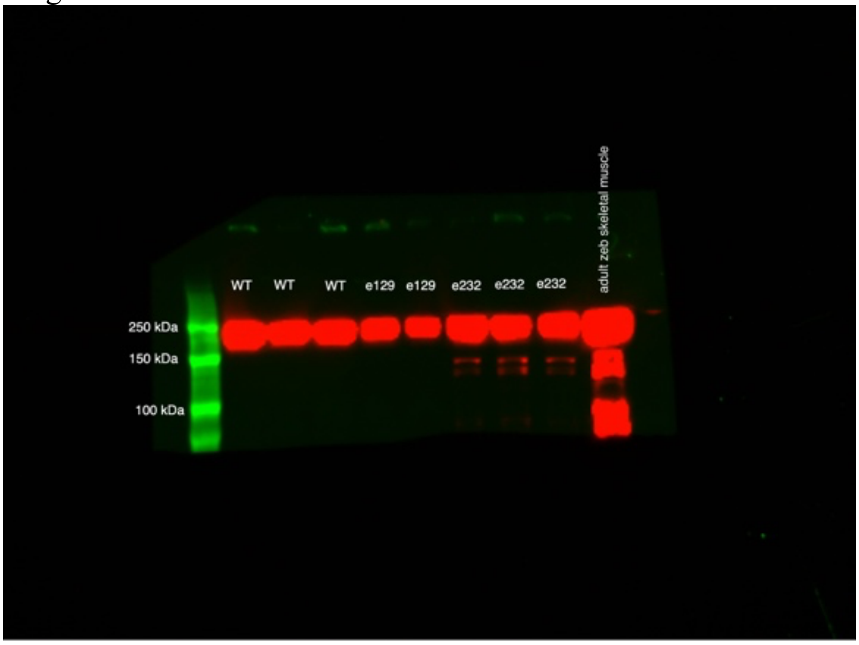

After conversion to B&W:

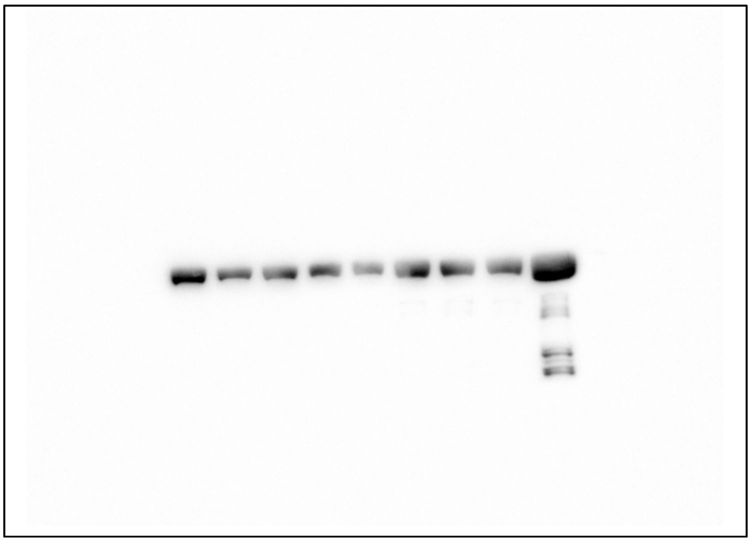

Full unedited blot for GAPDH Figure S9C:
Original:

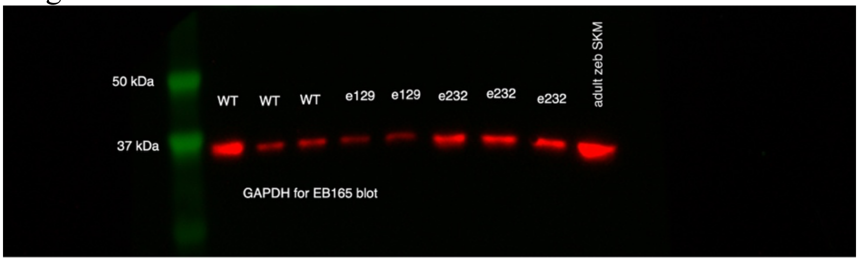

After conversion to B&W:

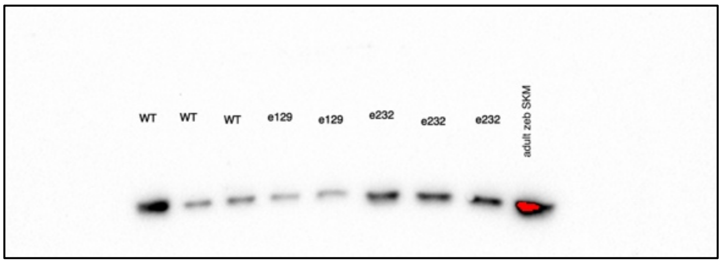

Supplement: Supplementary file 6 [file res-138-e325999-s006.pdf]
